# Natural locus coeruleus dynamics during feeding

**DOI:** 10.1126/sciadv.abn9134

**Published:** 2022-08-19

**Authors:** Natale R. Sciolino, Madeline Hsiang, Christopher M. Mazzone, Leslie R. Wilson, Nicholas W. Plummer, Jaisal Amin, Kathleen G. Smith, Christopher A. McGee, Sydney A. Fry, Cindy X. Yang, Jeanne M. Powell, Michael R. Bruchas, Alexxai V. Kravitz, Jesse D. Cushman, Michael J. Krashes, Guohong Cui, Patricia Jensen

**Affiliations:** ^1^ Neurobiology Laboratory, National Institute of Environmental Health Sciences, National Institutes of Health, Department of Health and Human Services, Research Triangle Park, NC, USA.; ^2^ Comparative Medicine, National Institute of Environmental Health Sciences, National Institutes of Health, Department of Health and Human Services, Research Triangle Park, NC, USA.; ^3^ Departments of Anesthesiology and Pharmacology, Center for the Neurobiology of Addiction, Pain, and Emotion, University of Washington, Seattle, WA, USA.; ^4^ Department of Psychiatry, Washington University, St. Louis, MO, USA.; ^5^ National Institute of Diabetes and Digestive and Kidney Diseases, National Institutes of Health, Department of Health and Human Services, Bethesda, MD, USA.

## Abstract

Recent data demonstrate that noradrenergic neurons of the locus coeruleus (LC-NE) are required for fear-induced suppression of feeding, but the role of endogenous LC-NE activity in natural, homeostatic feeding remains unclear. Here, we found that LC-NE activity was suppressed during food consumption, and the magnitude of this neural response was attenuated as mice consumed more pellets throughout the session, suggesting that LC responses to food are modulated by satiety state. Visual-evoked LC-NE activity was also attenuated in sated mice, suggesting that satiety state modulates LC-NE encoding of multiple behavioral states. We also found that food intake could be attenuated by brief or longer durations of LC-NE activation. Last, we found that activation of the LC to the lateral hypothalamus pathway suppresses feeding and enhances avoidance and anxiety-like responding. Our findings suggest that LC-NE neurons modulate feeding by integrating both external cues (e.g., anxiogenic environmental cues) and internal drives (e.g., satiety).

## Teaser

Dynamic activity of the locus coeruleus during feeding behavior is influenced by satiety state.

## INTRODUCTION

The ability of the brain to integrate internal physiological drives, such as hunger and satiety, with an ever-changing environment is essential for survival. It is well known that noradrenergic neurons of the locus coeruleus (LC-NE) play a key role in modulating diverse physiological and behavioral states such as arousal, sensory processing, stress, and attention (*1*–*3*). Upon the presentation of salient environmental stimuli, including nonnoxious and aversive events, LC-NE neurons are robustly activated (*4*–*7*). Much less is known about the role of LC-NE neurons in the regulation of feeding and the integration of internally driven motivational states, such as hunger and satiety.

Early electrophysiology studies in rats (*5*, *8*) and monkeys (*9*) noted inhibition of LC neurons during liquid consumption of sucrose or juice. In contrast, LC neurons are endogenously activated by cues predicting reward availability, and this activation is modulated by reward size and effort (*10*–*12*). Whether these endogenous LC responses play a causal role in the regulation of feeding behaviors remains unknown. Recently, it has been reported that chemogenetic activation of LC-NE neurons suppresses feeding in mice, and chemogenetic inhibition prevents fear-induced suppression of feeding (*13*). However, the role for LC-NE circuits in natural, homeostatic feeding remains unclear.

Investigation into the role of central NE circuits in feeding has primarily focused on medullary catecholamine neurons (*14*). Catecholamine neurons in the nucleus of the solitary tract (NTS) differentially modulate feeding in a circuit-dependent manner. Activation of an NTS projection to the arcuate stimulates feeding (*15*), while activation of an NTS projection to the parabrachial nucleus (PBN) suppresses feeding (*16*). In addition, activation of a ventrolateral medulla projection to the posterior paraventricular thalamus stimulates feeding and is required for glucoprivation-induced feeding (*17*). While it has been demonstrated that LC-NE neurons induce long-term depression at central amygdala synapses onto lateral PBN neurons in slice (*13*), it remains to be identified whether these circuits or others are involved in LC-NE regulation of feeding.

In the current manuscript, we combined in vivo fiber photometry calcium imaging, optogenetics, and chemogenetics with behavioral and metabolic approaches to demonstrate that the activity pattern of LC-NE neurons is enhanced during food approach and suppressed during feeding behavior in a manner influenced by satiety state. We also found that satiety state negatively influenced LC-NE activity response to visual sensory stimuli. In addition, we found that brief activation of LC-NE neurons during consumption plays a direct role in the suppression of feeding. Last, we found that the LC-lateral hypothalamus pathway suppresses feeding and elicits aversion and anxiety-like behavior.

## RESULTS

### LC-NE activity is suppressed during feeding in a manner influenced by satiety

**Fig. 1.**
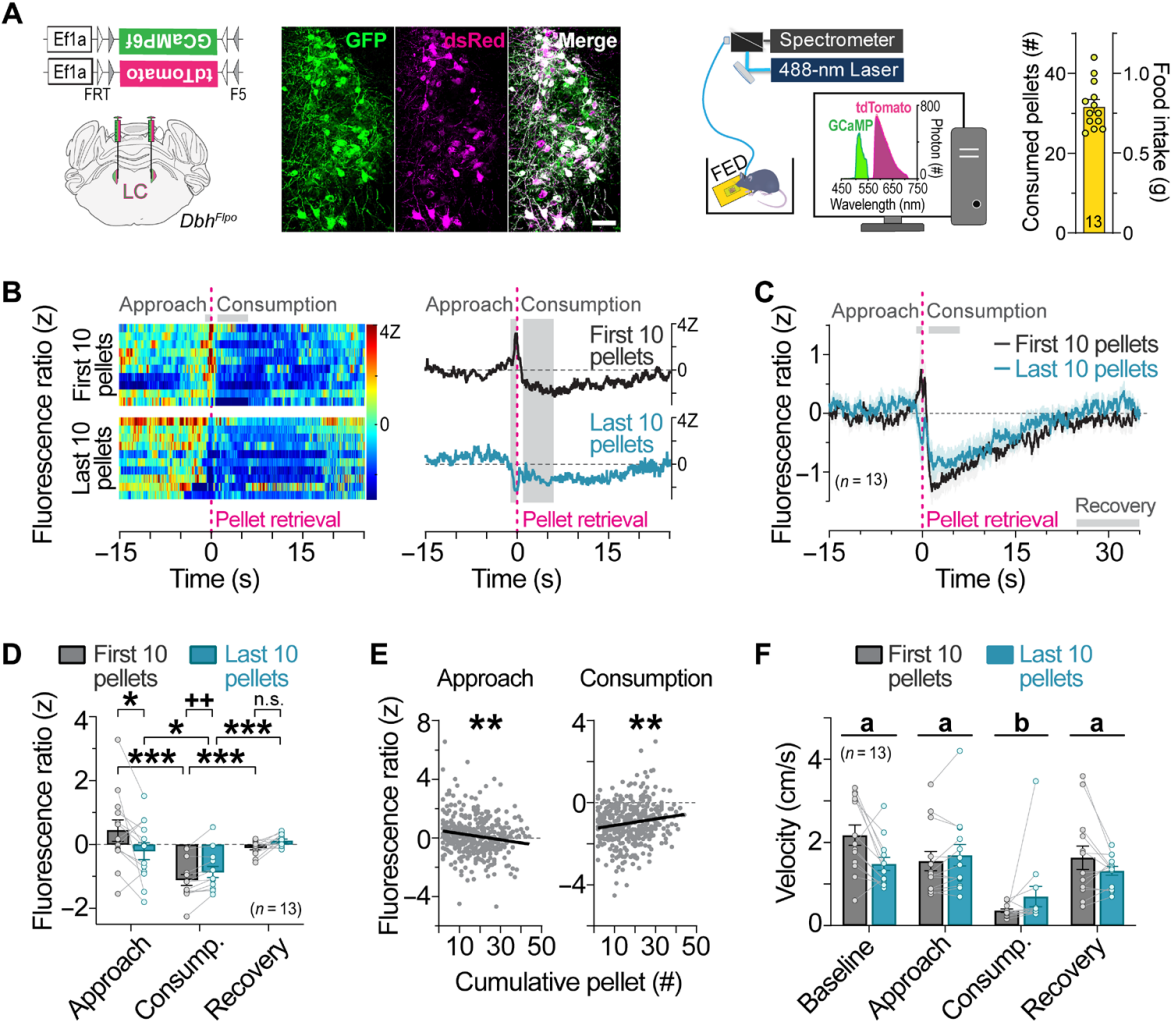
LC-NE activity is increased during food approach and suppressed during feeding in a manner influenced by satiety. (**A**) Left: Flp-dependent viral genetic strategy for coexpression of GCaMP6f and tdTomato (tdT) in LC-NE neurons. Middle: Coronal view of the locus coeruleus from an LC^GCaMP/tdT^ mouse immunostained for GCaMP6f (GFP antibody) and tdT (dsRed antibody). Scale bar, 50 μm. Right: Schematic of in vivo fiber photometry setup and feeding experimentation device (FED). Average food intake across the 1-hour session. Data are means ± SEM. *n* = 13 LC^GCaMP/tdT^ mice. (**B**) Fluorescence ratio expressed as a *z* score aligned to pellet retrieval during the first 10 pellets (top) and last 10 pellets (bottom) of the session from a representative fasted LC^GCaMP/tdT^ mouse. Single trials are represented in the heatmap (left), and average *z*-score fluorescence ratio is represented in the trace (right). (**C**) Average *z*-score fluorescence ratio aligned to pellet retrieval in fasted LC^GCaMP/tdT^ mice during the first 10 pellets and last 10 pellets of the session. Data are means ± SEM. *n* = 13 LC^GCaMP/tdT^ mice. (**D**) Average *z*-score fluorescence ratio during feeding-related behaviors. Two-way repeated-measures ANOVA, satiety × behavior interaction: *F*_2,24_ = 8.819, *P* = 0.0013. Bonferroni post hoc test, ***P < 0.001 and *P < 0.05. n.s., nonsignificant. Paired sample *t* test: *t*_12_ = 3.807, ^++^*P* < 0.01. Data are means ± SEM. *n* = 13 LC^GCaMP/tdT^ mice. (**E**) Linear regression of the average *z*-score fluorescence ratio during food approach and consumption in relation to pellet events across the sessions of fasted LC^GCaMP/tdT^ mice. Approach (slope = −0.02126 ± 0.006555; *R*^2^ = 0.02569; *F*_1,399_ = 10.52, **P < 0.01) and consumption (slope = 0.01562 ± 0.004806; *R*^2^ = 0.02579; *F*_1,399_ = 10.56, **P < 0.01). (**F**) Average velocity during feeding-related behaviors. Two-way repeated-measures ANOVA, main effect of behavior: *F*_3,36_ = 26.9, *P* < 0.001, satiety × behavior interaction: *F*_3,36_ = 4.457, *P* = 0.0092. Bonferroni post hoc test, *P* < 0.001 consumption (group b) versus all other behavioral states (group a). Data are means ± SEM. *n* = 13 LC^GCaMP/tdT^ mice.

To identify the natural activity patterns of LC-NE neurons during feeding, we used fiber photometry (*18*, *19*) to monitor fluorescent calcium activity using GCaMP6f in LC-NE neurons during food approach and consumption (Fig. 1A). Flp-dependent GCaMP6f and tdTomato (tdT) adenoassociated viruses (AAVs) were bilaterally injected into the LC (LC^GCaMP/tdT^) of mice expressing Flpo recombinase from the endogenous noradrenergic-specific dopamine-beta hydroxylase locus (*Dbh^Flpo^*) (Fig. 1A) (*20*). To minimize the effects of stress and novelty during the experiments, LC^GCaMP/tdT^ mice were habituated for several days to eat chow pellets from the feeding experimentation device (FED) (*21*). To determine whether hunger state modulates LC-NE neuronal responses to food, mice were fasted overnight, and unilateral LC responses were recorded as mice fed to satiety. Early in the session, we found that LC-NE neurons were activated during food approach and inhibited during consumption of pellets (Fig. 1, B to D), demonstrating a divergent pattern of LC activity during appetitive and consummatory behaviors. The approach-related LC-NE neuronal responses were completely abolished later in the session when mice had consumed more food, whereas the consummatory-related neuronal responses were slightly attenuated (Fig. 1, B to D). To determine whether transition from hunger to satiety had a gradual impact on food-related LC activity across the session, we next performed linear regression analyses. We found that LC-NE neuronal responses during approach and consumption were gradually attenuated with successive pellets in the session (Fig. 1E). We observed no sex differences in LC-NE activity to food (fig. S1). Further, velocity remained stable as satiation developed across the session (Fig. 1F), indicating that ambulation did not drive satiety-related changes in LC-NE activity during feeding. Together, our findings demonstrate that endogenous activity of LC-NE neurons is enhanced during food approach and suppressed during food consumption, and that these food-evoked responses are attenuated by satiety.

### Visual-evoked LC-NE activity is attenuated in sated mice

**Fig. 2.**
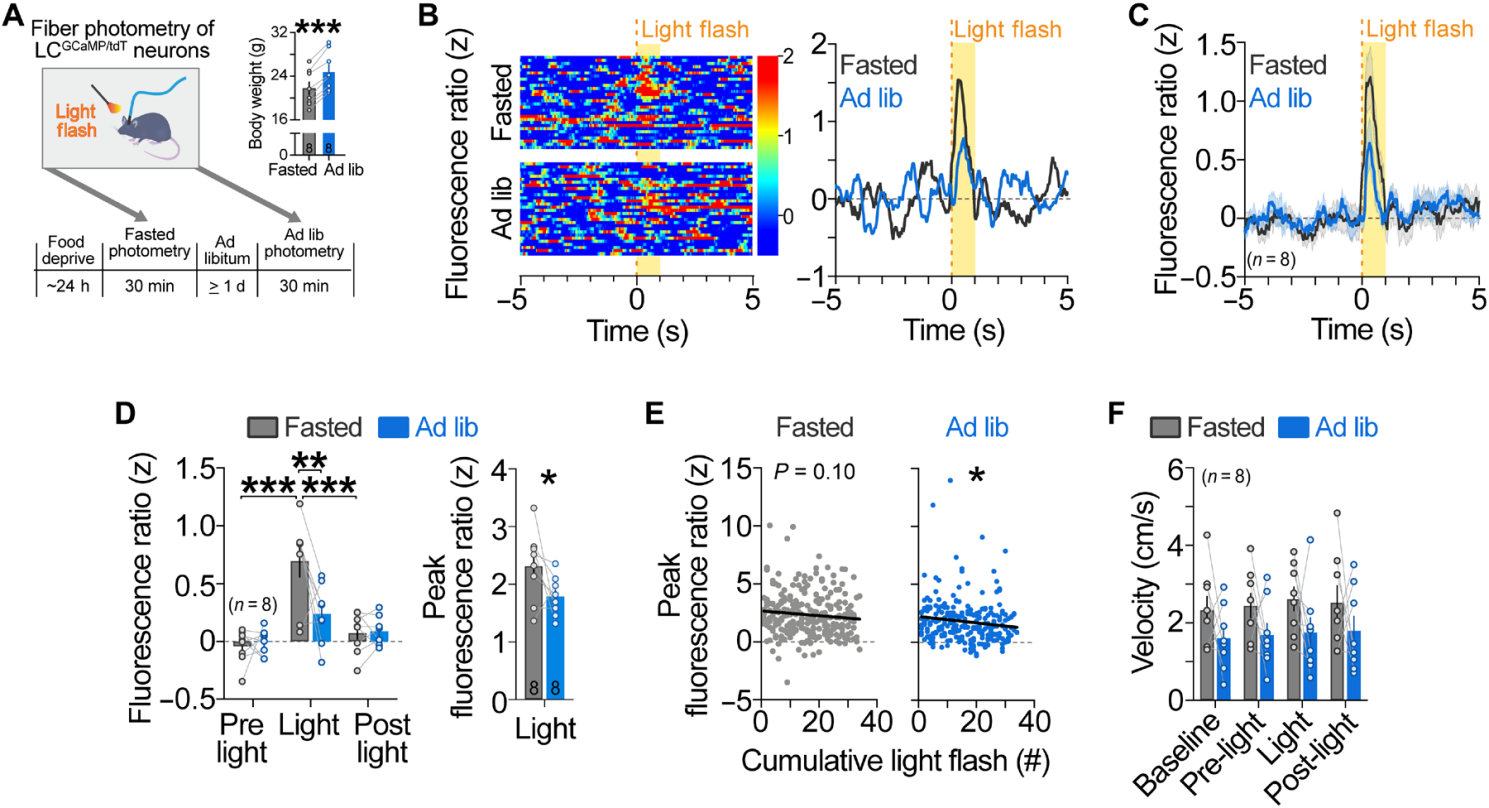
Visual-evoked LC-NE activity is influenced by satiety. (**A**) Left: Timeline of photometry recordings during presentation of light flashes. Right: Body weights during fasted and ad libitum–fed recordings. Paired samples *t* test, ***P < 0.001. Data are means ± SEM. *n* = 8 LC^GCaMP/tdT^ mice. (**B**) Fluorescence ratio expressed as a *z* score aligned to visual stimulus during the fasted (top) and ad libitum–fed (bottom) recordings from a representative LC^GCaMP/tdT^ mouse. Single trials are represented in the heatmap (left), and average *z*-score fluorescence ratio is represented in the trace (right). (**C**) Average *z*-score fluorescence ratio aligned to visual stimulus. (**D**) Left: Average *z*-score fluorescence ratio during visual-related events in LC^GCaMP/tdT^ mice. Two-way repeated-measures ANOVA, satiety × event interaction: *F*_2,14_ = 8.681, *P* = 0.0035. Bonferroni post hoc test, ***P < 0.001 and **P < 0.01. Right: Average peak *z*-score fluorescence ratio during visual events. Paired sample *t* test (one-tailed): *t*_7_ = 1.942, *P* = 0.0466. Data are means ± SEM. *n* = 8 LC^GCaMP/tdT^ mice. (**E**) Linear regression of the average peak *z*-score fluorescence ratio during light flashes across the fasted and ad libitum–fed sessions of LC^GCaMP/tdT^ mice. Fasted (slope = −0.02061 ± 0.01245; *R*^2^ = 0.01055, *F*_1,257_ = 2.740, *P* = 0.0991) and ad libitum–fed (slope = −0.02777 ± 0.01238; *R*^2^ = 0.01949; *F*_1,253_ = 5.028, *P* = 0.0258). (**F**) Average velocity during visual-related events. Two-way repeated-measures ANOVA, main effect of satiety: *F*_1,7_ = 1.728, *P* = 0.2301; main effect of event: *F*_3,21_ = 2.235, *P* = 0.1140; satiety × event interaction: *F*_3,21_ = 0.1346, *P* = 0.9383. Data are means ± SEM. *n* = 8 LC^GCaMP/tdT^ mice.

Next, we wanted to determine whether satiety state affects LC-NE responses to nonfood stimuli. It is well established that LC neurons are activated by salient or unexpected sensory events (*4*, *5*), but whether this response is modulated by internal physiological states such as satiety is unknown. To address this question, we used fiber photometry to compare LC-NE activity evoked by a salient, 1-s flash of light presented to mice that were fasted overnight or fed ad libitum (Fig. 2A). In fasted LC^GCaMP/tdT^ mice, we observed a rapid, transient activation of LC-NE neurons to the visual stimulus (Fig. 2, B to D). In mice fed ad libitum, however, the magnitude of LC-NE activity was smaller (Fig. 2, B to D), demonstrating that satiation attenuated LC-NE responses to visual stimuli. To determine whether LC response to visual stimuli habituated across successive presentations, we next performed linear regression analyses on peak fluorescence ratio across the session for fasted and ad libitum–fed mice. The peak in activity of LC-NE neurons induced by the light flash was significantly attenuated across the repeated trials in ad libitum–fed mice, with a similar trend for an attenuated response in fasted mice (Fig. 2E), consistent with prior electrophysiology studies in rat that have noted that LC activation to the visual stimulus habituates across repeated presentations (*4*, *5*). We observed no sex differences in LC-NE activity to light flashes (fig. S2). Further, movement velocity remained constant during visual stimulus presentation for fasted and sated mice (Fig. 2F), indicating that ambulation did not drive visual-evoked activity of LC neurons. Collectively, our results demonstrate that internal satiety state affects LC-NE response to both food-related and non–food-related stimuli.

### Brief, behaviorally paired activation of LC-NE neurons suppresses feeding

**Fig. 3.**
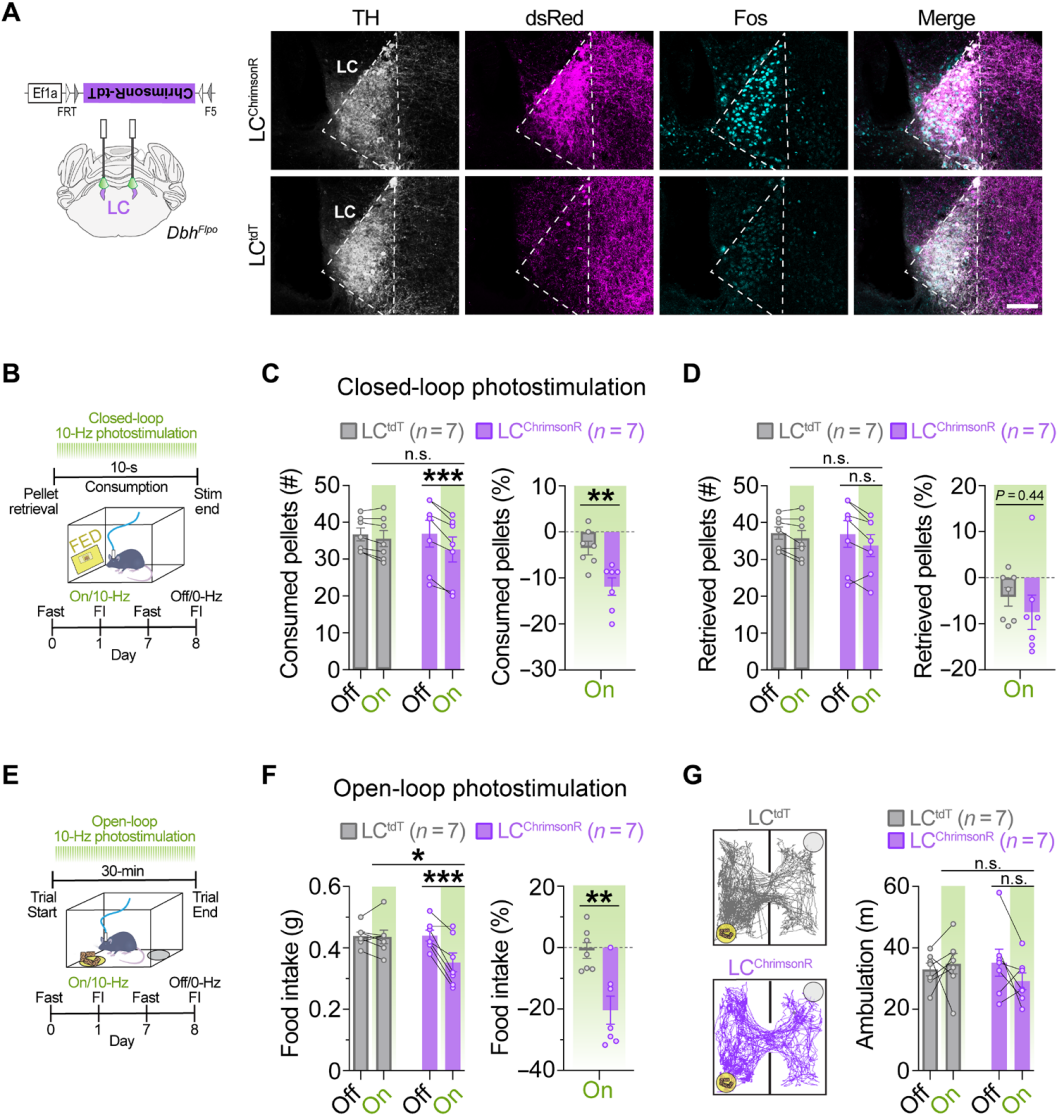
Optogenetic stimulation of LC-NE cell bodies suppresses feeding. (**A**) Left: Schematic of coronal mouse brain showing location of Flp-dependent AAV to drive ChrimsonR-tdT expression. Right: Immunofluorescent labeling of Fos (turquoise) in noradrenergic (TH, light gray), tdT-expressing (dsRed, magenta) locus coeruleus neurons in coronal brain sections from a representative LC^tdT^ and LC^ChrimsonR^ mouse following photostimulation (560 nm, 10 Hz, 10 ms) for 30 min. Scale bar, 100 μm. (**B**) Timeline of closed-loop optogenetic experiment wherein photostimulation (10 Hz) was triggered upon pellet retrieval and occurred briefly for 10 s during consumption from the FED. (**C** and **D**) Left: Average feeding behavior of fasted mice during closed-loop optogenetic experiment. Two-way repeated-measures ANOVA, stimulation × virus interaction: number of pellets consumed (C; *F*_1,12_ = 13.70, *P* = 0.0030) and pellets retrieved (D; *F*_1,12_ = 1.571, *P* = 0.2339). Bonferroni post hoc test, ***P < 0.001 and **P < 0.01. Data are means ± SEM. *n* = 7 LC^tdT^ mice, *n* = 7 LC^ChrimsonR^ mice. Right: Feeding behavior during closed-loop photostimulation <<(On) as percent change from no stimulation (Off) in fasted mice. Unpaired sample *t* test: percent change in pellets consumed (C; *t*_12_ = 3.504, **P < 0.01) and pellets retrieved (D; *t*_12_ = 0.7904, *P* = 0.4446). (**E**) Timeline of food intake (FI) during open-loop photostimulation (10 Hz, 10-ms pulses). (**F**) Left: Average 30-min food intake during the presence (On) or absence (Off) of open-loop photostimulation in fasted mice. Two-way repeated-measures ANOVA, stimulation × virus interaction: *F*_1,12_ = 14.42, *P* = 0.0025. Bonferroni post hoc test, ***P < 0.001, **P < 0.01, and *P < 0.05. Right: Food intake during photostimulation as percent change from no photostimulation. Unpaired sample *t* test, *t*_12_ = 3.716, **P < 0.01. (**G**) Ambulation in FI task during open-loop photostimulation. Left: Representative traces show ambulation, yellow circle indicates food cup location, and gray circle indicates empty cup location. Right: Average ambulation in FI task in fasted mice. Two-way repeated-measures ANOVA, stimulation × virus interaction: *F*_1,12_ = 2.149, *P* = 0.1684. Data are means ± SEM. *n* = 7 LC^tdT^ mice and *n* = 7 LC^ChrimsonR^ mice.

Given our finding that endogenous activity of LC-NE neurons is attenuated during feeding, we next sought to determine whether feeding would be suppressed by brief stimulation of LC-NE neurons during the 10-s of feeding immediately following pellet retrieval. To test this hypothesis, we injected Flp-dependent AAVs expressing the red-shifted channelrhodopsin variant ChrimsonR-tdT (*22*, *23*) or tdT control in the LC of *Dbh^Flpo^* mice (Fig. 3A) (*20*). LC^ChrimsonR^ and LC^tdT^ mice were trained to eat grain pellets from the FED, fasted overnight, and then behaviorally monitored, while brief photostimulation was paired in real-time with feeding (Fig. 3B). We found that LC^ChrimsonR^ mice consumed less during brief photostimulation compared to controls (Fig. 3C). LC^ChrimsonR^ mice dropped more pellets and retrieved less pellets during brief photostimulation, but the difference compared to controls was not statistically significant (*P* = 0.07 and *P* = 0.44; fig. S3A and Fig. 3D), suggesting that stimulating LC-NE neurons redirects focus away from feeding. We observed no sex differences in feeding suppression induced by optogenetic stimulation of LC-NE neurons (fig. S4A). These findings demonstrate that brief, behaviorally locked activation of LC-NE neurons is sufficient to suppress feeding in hungry mice, a response that may be due to reduced attention/distractibility that causes the animal to disengage from its current behavior.

Recently, it has been shown that chemogenetic activation of LC-NE neurons suppresses feeding (*13*). To determine whether we could replicate this effect using a longer duration of optogenetic stimulation, LC^ChrimsonR^ and LC^tdT^ control mice were overnight fasted, and intake of standard chow was measured for 30 min in the presence or absence of 560-nm optical pulses (10 Hz, 10 ms) (Fig. 3E). We found that LC^ChrimsonR^ mice had reduced food intake (FI) during optical stimulation compared to controls (Fig. 3F), without affecting ambulation (Fig. 3G), indicating that LC-mediated suppression of feeding can occur independently from changes in ambulation. Given our finding that endogenous activity of LC-NE neurons is increased during food approach, we next sought to determine whether food seeking behaviors would be increased in LC^ChrimsonR^ mice using the longer duration of optogenetic stimulation. During photostimulation, we found that LC^ChrimsonR^ mice tended to both enter the food zone more frequently and spend more time in the food zone compared to no stimulation (fig. S3B), suggesting that longer-term stimulation of LC-NE neurons could have a limited effect on approach behavior. Together, our findings demonstrate that feeding can be suppressed by brief, behaviorally locked activation of LC-NE neurons and longer durations of optogenetic stimulation.

### Activation of the LC-NE suppresses feeding and promotes weight loss without affecting metabolism

**Fig. 4.**
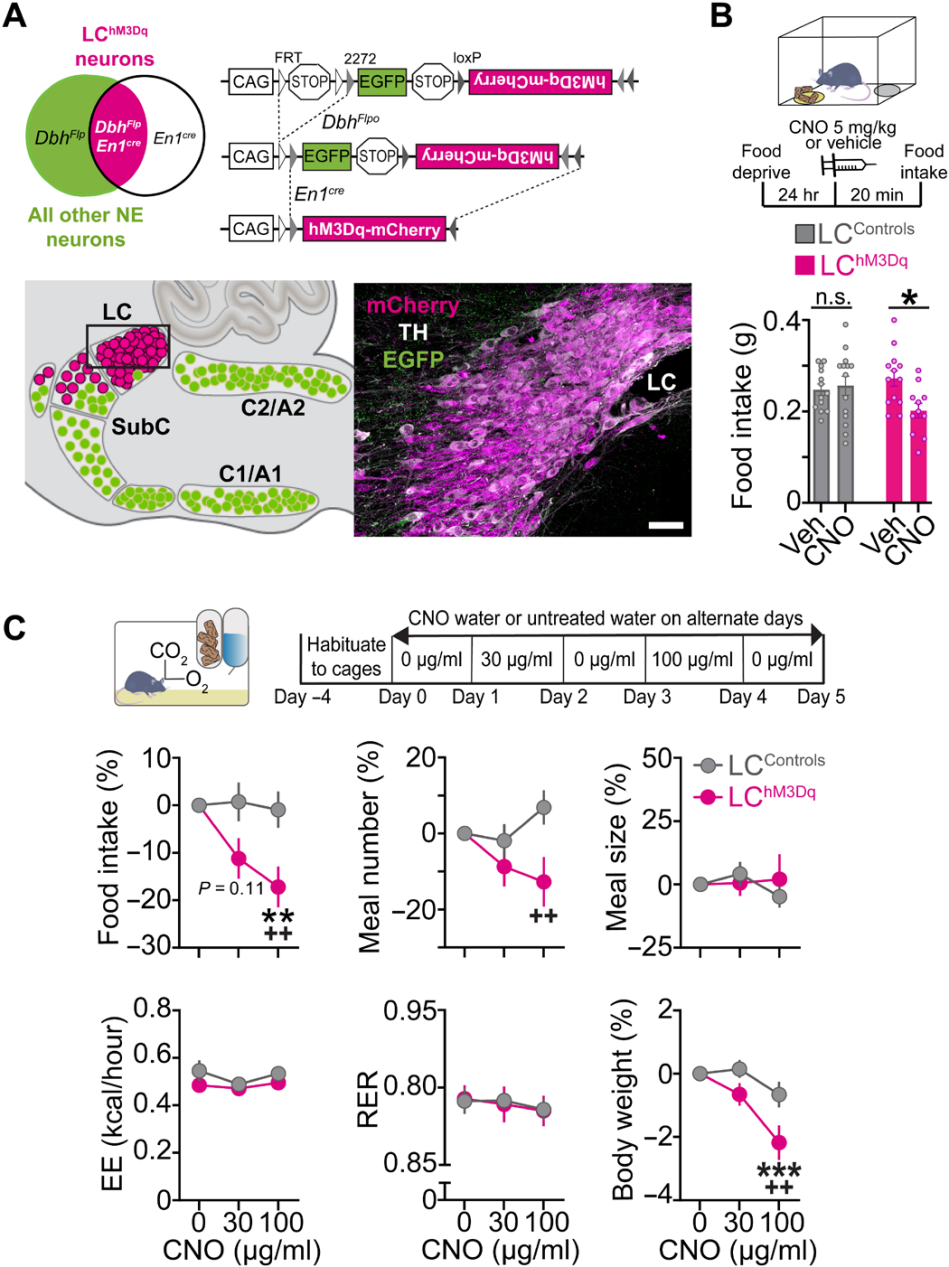
Chemogenetic activation of LC-NE neurons suppresses feeding without altering metabolism. (**A**) Left: Schematic illustration of intersectional genetic strategy. Recombination of *RC::FL-hM3Dq* allele by *Dbh^Flpo^* and *En1^cre^* results in hM3Dq-mCherry expression in LC-NE neurons. Recombination by *Dbh^Flpo^* alone leads to EGFP expression. Right: Schematic of sagittal mouse hindbrain compressed across the mediolateral axis. Parasagittal section from LC^hM3Dq^ brain reveals hM3Dq-mCherry expression in LC-NE neurons. Scale bar, 50 μm. (**B**) Top: Timeline of FI experiments in fasted mice. Bottom: Average FI in fasted mice. Two-way between-subject ANOVA, drug × genotype interaction: *F*_1,47_ = 5.20, *P* = 0.0272. Bonferroni post hoc test, *P < 0.05. Data are means ± SEM. *n* = 13 vehicle-treated and *n* = 14 CNO-treated LC^Controls^. *n* = 13 vehicle-treated LC^hM3Dq^ mice, *n* = 11 CNO-treated LC^hM3Dq^ mice. (**C**) Top: Timeline of CNO water at 30 and 100 μg/ml. Bottom: Behavioral and metabolic measures in the automated homecage. Two-way repeated-measures ANOVA, drug × genotype interaction: food intake (*F*_2,54_ = 3.64, *P* = 0.0329), meal number (*F*_2,54_ = 3.478, *P* = 0.0379), meal size (*F*_2,54_ = 0.8542, *P* = 0.4313), energy expenditure (EE; *F*_2,54_ = 0.4781, *P* = 0.6226), respiratory exchange rate (RER; *F*_2,54_ = 0.04714, *P* = 0.9540), and body weight (*F*_2,70_ = 3.647, *P* = 0.0312). Bonferroni post hoc test, ***P < 0.001 and **P < 0.01 versus vehicle; ^++^*P* < 0.01 versus LC^Controls^. Data are means ± SEM. *n* = 20 LC^Controls^ and *n* = 9 LC^hM3Dq^ mice for all measures except body weight wherein *n* = 17 LC^hM3Dq^ mice.

To determine the metabolic effects of activating LC-NE neurons, we used triple transgenic *En1^cre^; Dbh^Flpo^; RC::FL-hM3Dq* (LC^hM3Dq^) mice expressing the excitatory Gq-coupled receptor hM3Dq fused to mCherry (Fig. 4A) (*24*). This previously published nonviral intersectional approach allows selective and reproducible expression of hM3Dq in 99.6% of the anatomically defined LC located within the central gray (*25*), and a small portion of the dorsal subcoeruleus and A7 immediately adjacent to and continuous with the LC (*20*, *24*, *26*–*28*). To add to our prior characterization of the LC^hM3Dq^ mouse line, we first performed in vivo fiber photometry in LC^hM3Dq^ mice expressing GCaMP6f and littermate control mice expressing GCaMP6f (LC^Controls/GCaMP6f^) or lacking GCaMP6f expression (LC^Controls/EGFP^) following administration of clozapine *N*-oxide (CNO; 5 mg/kg, intraperitoneally) or vehicle. Recordings revealed a sustained increase in LC-NE activity following CNO treatment in LC^hM3Dq^ mice compared to controls (fig. S5). No change in fluorescence was observed in LC^Controls/GCaMP6f^ mice and LC^Controls/EGFP^ littermate controls after treatment with CNO or vehicle (fig. S5). In all mice expressing GCaMP6f, but not green fluorescent protein (GFP), we observed increases in LC activity during handling and injection (fig. S5), validating the sensitivity of our recording conditions to detect an established threat-related response (*4*, *29*, *30*).

To confirm that our chemogenetic strategy for activation of LC-NE neurons would suppress feeding in hungry mice, LC^hM3Dq^ mice and littermate controls were overnight fasted and given an intraperitoneal dose of CNO or vehicle before feeding was measured in a novel arena (Fig. 4B). We found that CNO suppressed feeding in LC^hM3Dq^ mice, but not littermate controls (Fig. 4B), demonstrating that chemogenetic activation of LC-NE neurons suppresses feeding in hungry mice, consistent with published results (*13*). To determine precisely how LC activation influences daily feeding and metabolism, and to rule out novelty-induced suppression of feeding as a variable, LC^hM3Dq^ and littermate control mice were housed for several days in the Labmaster homecage, which tracks meals and metabolic parameters by indirect calorimetry (energy expenditure and respiratory exchange rate) (Fig. 4C). To avoid the stress induced by daily injection of CNO, plain water or CNO (30 or 100 μg/ml) in the drinking water was administered on alternate days. In CNO-treated LC^hM3Dq^ mice, activation of LC-NE neurons dose-dependently suppressed feeding by reducing the number of meals consumed, without affecting meal size (Fig. 4C and fig. S6). These changes resulted in weight loss in CNO-treated LC^hM3Dq^ mice (Fig. 4C). Analysis of circadian behavior revealed that suppression of feeding and drinking occurred specifically during lights off (when LC^hM3Dq^ mice drank the most CNO), and this effect was reversible during lights on (when mice drank less CNO) and upon removal of the drug the next day (fig. S7), indicating that LC-mediated suppression of ingestive behavior was specific and reversible. Further, using indirect calorimetry, we found that CNO had no effect on energy expenditure or respiratory exchange rate in LC^hM3Dq^ mice and littermate controls (Fig. 4C). The average daily dose of CNO was similar for LC^hM3Dq^ mice and littermate controls (fig. S7C), although females drank more CNO water regardless of genotype (fig. S8A). This larger daily dose of CNO water may explain the trend for a greater food suppression and weight loss in female LC^hM3Dq^ mice compared to male controls (fig. S8B). These findings collectively demonstrate that activation of LC-NE neurons suppresses feeding without altering metabolism, and therefore, we focused our efforts to measures of food intake in subsequent experiments.

### Acute inhibition of LC-NE neurons has no significant effect on food intake

Given that LC-NE activation suppresses feeding, we next sought to determine whether inhibition of LC-NE neurons would promote feeding. To test this hypothesis, we injected a cre-dependent AAV expressing the Gi-coupled receptor hM4Di-mCherry (*31*) or mCherry in the LC of *Dbh^cre^* mice (fig. S9A) (*32*). To confirm that the expressed hM4Di receptor was functional, we replicated a prior chemogenetic study (*33*) and found that inhibiting LC-NE neurons attenuates restraint stress-induced anxiety-like behavior in the open field, without affecting total ambulation (fig. S9B). To determine whether LC-NE activity is required for feeding, LC^hM4Di^ mice and controls were overnight fasted and treated with CNO (1 mg/kg, intraperitoneally) or vehicle before placement in a familiar arena containing standard chow (fig. S9C). No significant change in feeding was observed between CNO-treated LC^hM4Di^ mice and LC^mCherry^ controls (fig. S9C), suggesting that LC-NE activity is not necessary for promoting feeding in hungry mice.

### Stimulation of LC-lateral hypothalamus circuit suppresses feeding, elicits aversion, and enhances anxiety-like behavior

Prior studies have shown that LC-NE neurons project to the lateral hypothalamus area (LHA) (*20*, *33*, *34*) and that NE has a strong inhibitory effect when applied directly in the LHA (*35*–*39*). To determine whether the LC may mediate this effect, we measured Fos immunoreactivity in LC^hM3Dq^ mice following treatment of CNO (1 mg/kg, intraperitoneally) or vehicle. Activation of LC-NE neurons resulted in a significant reduction in Fos expression in the LHA (fig. S10, A and C). To determine whether this response was specific to the LHA, we measured Fos expression in two additional feeding-related targets of the LC, the dorsal medial and ventromedial hypothalamic nuclei. We observed no change in Fos expression in either nucleus (fig. S10, B and C).

**Fig. 5.**
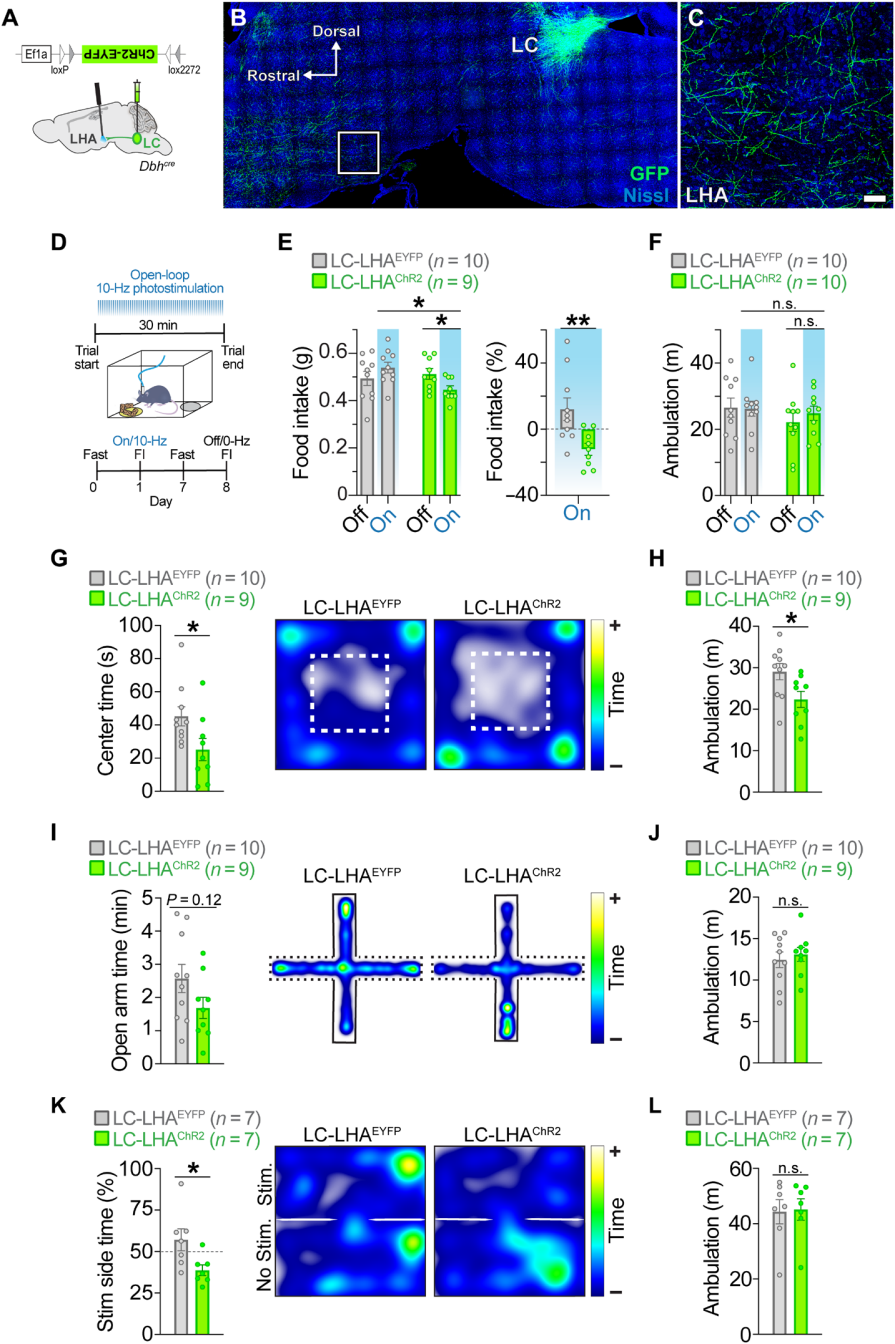
Optogenetic stimulation of the LC-LHA noradrenergic pathway suppresses feeding, elicits aversion, and enhances anxiety-like behavior. (**A**) Schematic illustration of sagittal mouse brain shows location of cre-dependent AAV used to drive ChR2-EYFP expression and location of fiber-optic probes. (**B** and **C**) Parasagittal brain section from a *Dbh^cre^;* LC-LHA^EYFP^ mouse shows restricted EYFP expression in LC-NE neurons. High-magnification image shows EYFP-expressing LC-NE axonal projections in the LHA. Scale bars, 400 μm (brain) and 150 μm (LHA). (**D**) Timeline of FI during open-loop photostimulation (465 nm, 10 Hz, 10-ms pulses). (**E** and **F**) Average feeding-related behaviors measured in the presence (On) or absence (Off) of open-loop photostimulation in fasted mice. Two-way repeated-measures ANOVA, stimulation × virus interaction: food intake (E, left; *F*_1,17_ = 10.8, *P* = 0.0044) and ambulation (F; *F*_1,18_ = 0.655, *P* = 0.4290). Bonferroni post hoc test, *P < 0.05. Data are means ± SEM. *n* = 10 LC-LHA^EYFP^ mice, *n* = 9 to 10 LC-LHA^ChR2^ mice. Unpaired sample *t* test: food intake during photostimulation as a percent change from no photostimulation (E, right; *t*_17_ = 3.055, **P < 0.01). Data are means ± SEM. *n* = 10 LC-LHA^EYFP^ mice and *n* = 9 LC-LHA^ChR2^ mice. (**G** and **H**) OFT behaviors. Unpaired sample *t* test: center time (G; *t*_17_ = 2.279, *P < 0.05) and ambulation (H; *t*_17_ = 2.411, *P < 0.05). Data are means ± SEM. *n* = 10 LC-LHA^EYFP^ mice and *n* = 9 LC-LHA^ChR2^ mice. (**I** and **J**) EPM behaviors. Unpaired sample *t* test: open arm time (I; *t*_17_ = 1.638, *P* = 0.1199) and ambulation (J; *t*_17_ = 0.5101, *P* = 0.6165). Data are means ± SEM. *n* = 10 LC-LHA^EYFP^ mice and *n* = 9 LC-LHA^ChR2^ mice. (**K** and **L**) RTPT behaviors. Unpaired sample *t* test: stimulation side time (K; *t*_12_ = 2.498, *P < 0.05) and ambulation (L; *t*_12_ = 0.1369, *P* = 0.8934). Data are means ± SEM. *n* = 7 LC-LHA^EYFP^ mice and *n* = 7 LC-LHA^ChR2^ mice. (G, I, and K) Representative spatial location heatmaps show time spent exploring the arenas.

To directly test whether stimulation of the LC-LHA circuit suppresses feeding, we injected AAVs expressing cre-dependent channelrhodopsin-2 (ChR2) (*40*, *41*) or enhanced yellow fluorescent protein (EYFP) in the LC of *Dbh^cre^* mice (Fig. 5A) (*32*). Consistent with prior observations (*20*, *33*, *34*), we observed LC-derived axons in the LHA (Fig. 5, B and C). We next measured food intake of overnight fasted LC-LHA^ChR2^ and control mice in the presence or absence of 465-nm optical pulses (10 Hz, 10 ms) for 30 min while in a familiar arena (Fig. 5D). Optical stimulation suppressed feeding in LC-LHA^ChR2^ mice without affecting ambulation (Fig. 5, E and F). No change in these behaviors was observed in LC-LHA^EYFP^ controls following optical stimulation (Fig. 5, E and F). Our findings demonstrate that activation of the LC-LHA noradrenergic pathway is sufficient to suppress feeding in hungry mice.

We next sought to determine whether stimulating the LC-LHA pathway would elicit negative affective behaviors associated with stress, as it is well known that stressful events activate LC-NE neurons (*42*, *43*), and stimulating these neurons mimics stress by producing anxiety and aversion (*24*, *33*, *44*). To measure anxiety-like behavior, we ran LC-LHA^ChR2^ and LC-LHA^EYFP^ mice in the open field test (OFT) and elevated plus maze (EPM) during optical stimulation. We observed that LC-LHA^ChR2^ mice spent significantly less time in the center of the OFT (Fig. 5G) and tended to spend less time in the open arms of the EPM (Fig. 5I), demonstrating that activation of the LC-LHA noradrenergic pathway is anxiogenic. To assess whether stimulation of the LC-LHA pathway has a negative or positive valence, we used a real-time place preference test (RTPT) that triggers photostimulation upon entry into a designated side of the arena. We found that LC-LHA^ChR2^ mice spent less time in the stimulation-paired side compared to LC-LHA^EYFP^ controls (Fig. 5K), indicating an aversive behavioral response resulting from LC-LHA circuit activation. During photostimulation, LC-LHA^ChR2^ mice had reduced ambulation in the OFT compared to controls, but no change in the EPM and RTPT (Fig. 5, H, J, and L), suggesting that the LC-LHA circuit does not have an overall impact on locomotion. Further, male and female mice had a similar magnitude of suppressed feeding as well as enhanced anxiety-like and aversive responses following optogenetic stimulation of the LC-LHA pathway (fig. S11). Subsequent assessment of Fos immunoreactivity revealed that photostimulation of ChR2-expressing LHA terminals did not induce antidromic activity of LC-NE neurons (fig. S12). These findings demonstrate that activating the LC-LHA pathway elicits negative affect behaviors and suppresses feeding when photostimulation is pulsed throughout the behavioral paradigm.

### Stimulation of LC-LHA circuit suppresses feeding when stimulation is pulsed for longer durations, not when briefly paired with feeding

**Fig. 6.**
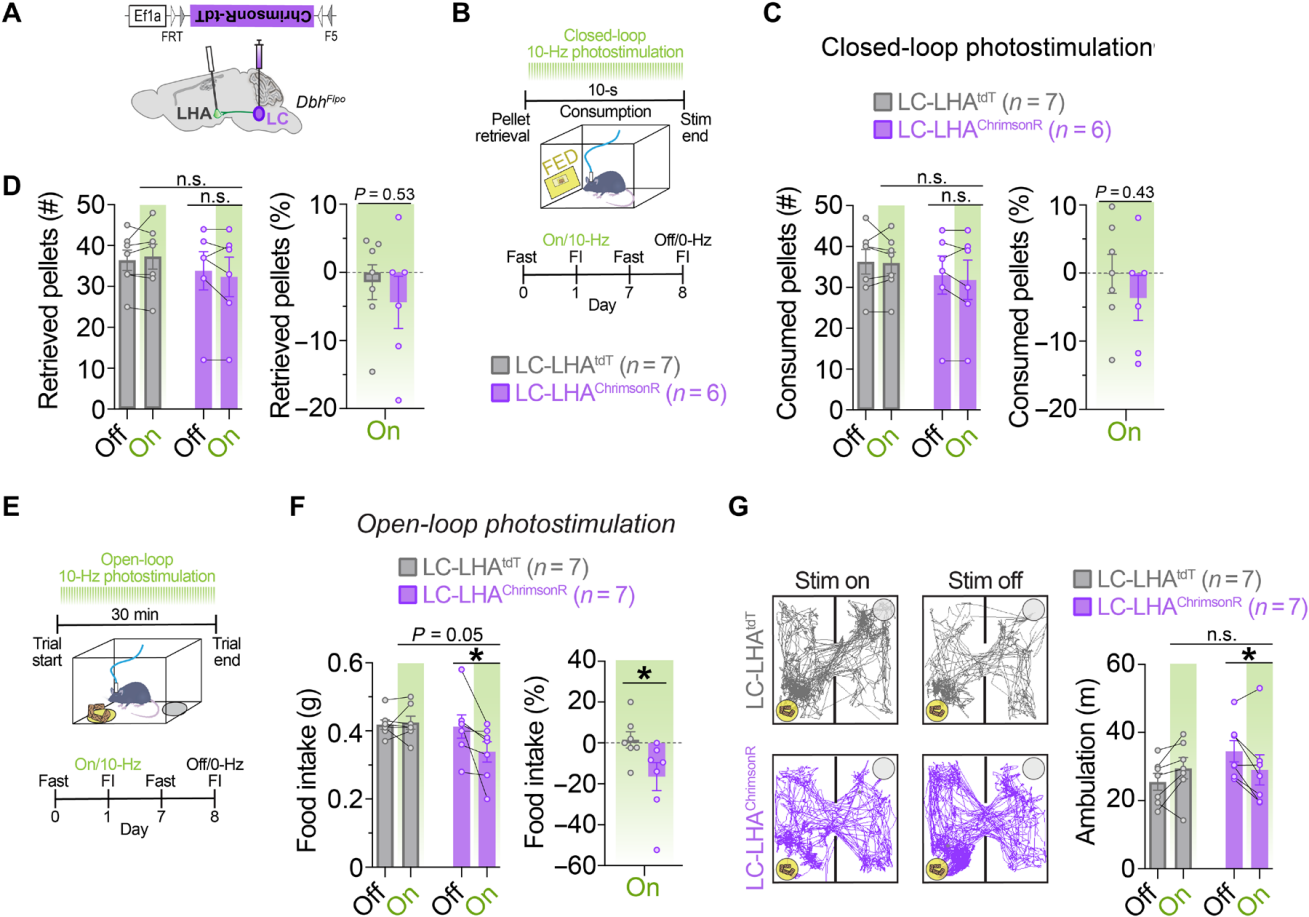
Optogenetic stimulation of the LC-LHA noradrenergic pathway suppresses feeding when stimulation is pulsed during the entire behavioral assay, but not when restricted to feeding events. (**A**) Schematic illustration of coronal mouse brain shows location of Flp-dependent AAV used to drive ChrimsonR-tdT expression and location of fiber-optic probes. (**B**) Timeline of closed-loop optogenetic experiment wherein photostimulation (560 nm, 10 Hz, 10-ms pulses) was triggered upon pellet retrieval and occurred briefly for 10 s during consumption from the FED. (**C** and **D**) Left: Average feeding behavior of fasted mice during the closed-loop optogenetic experiment. Two-way repeated-measures ANOVA, stimulation × virus interaction: number of pellets consumed (C; *F*_1,11_ = 0.2807, *P* = 0.6068) and pellets retrieved (D; *F*_1,11_ = 1.857, *P* = 0.2003). Data are means ± SEM. *n* = 7 LC-LHA^tdT^ mice and *n* = 6 LC-LHA^ChrimsonR^ mice. Right: Feeding behavior of fasted mice during closed-loop optogenetic experiment as percent change of photostimulation (On) from no stimulation (Off). Unpaired sample *t* test: percent change in pellets consumed (C; *t*_11_ = 0.8160, *P* = 0.4318) and pellets retrieved (D; *t*_11_ = 0.6549, *P* = 0.5260). (**E**) Timeline of FI during open-loop photostimulation (10 Hz, 10-ms pulses). (**F**) Left: Average 30-min food intake of fasted mice in the presence (On) or absence (Off) of open-loop photostimulation. Two-way repeated-measures ANOVA, stimulation × virus interaction: *F*_1,12_ = 5.116, *P* = 0.0431. Bonferroni post hoc test, *P < 0.05. Data are means ± SEM. *n* = 7 LC-LHA^tdT^ mice and *n* = 7 LC-LHA^ChrimsonR^ mice. Right: Food intake during photostimulation (On) as a percent change from no photostimulation (Off). Unpaired sample *t* test, *t*_12_ = 2.269, *P < 0.05. (**G**) Ambulation in FI task during open-loop photostimulation. Left: Representative traces show ambulation, yellow circle indicates food cup location, and gray circle indicates location of an empty cup. Right: Average 30-min ambulation of fasted mice in FI assay during open-loop photostimulation. Two-way repeated-measures ANOVA, stimulation × virus interaction: *F*_1,12_ = 10.33, *P* = 0.0074. Bonferroni post hoc test, *P < 0.05.

To test whether feeding would be suppressed by selective activation of the LC-LHA pathway during consumption, we injected AAVs expressing Flp-dependent ChrimsonR-tdT (*22*, *23*) or tdT in the LC of *Dbh^Flpo^* mice (Fig. 6A). LC-LHA^ChrimsonR^ mice and controls were trained to eat grain-based pellets from the FED and overnight fasted, and feeding was measured while 560-nm optical pulses (10 Hz, 10 ms) were delivered for 10 s during consumption (Fig. 6B), when LC-NE neurons are endogenously less active (Fig. 1, B to D). We observed no significant change in the number of pellets consumed or retrieved between LC-LHA^ChrimsonR^ mice and controls (Fig. 6, C and D), suggesting that feeding is unaffected by brief, behaviorally locked activation of the LC-LHA pathway. In a separate experiment delivering 30 min of stimulation (10-Hz, 10-ms), we found that this longer duration of pulsing suppressed food intake and ambulation in LC-LHA^ChrimsonR^ mice (Fig. 6, E to G), effectively reproducing the feeding suppression observed in LC-LHA^ChR2^ mice (Fig. 5E). Further, male and female mice had a similar magnitude of feeding suppression following optogenetic stimulation of the LC-LHA circuit (fig. S4B). Together, our findings demonstrate that stimulating the LC-LHA pathway for longer durations suppresses feeding potentially due to an enhancement of negative valence and reduction in locomotor activity.

### Acute inhibition of the LC-LHA pathway has no significant effect on food intake

To test whether inhibition of the LC-LHA circuit enhances feeding, *Dbh^cre^* mice^(^*^32^*^)^ were injected with AAVs expressing a cre-dependent hM4Di (*31*) receptor or mCherry and implanted with bilateral cannula above the LHA (fig. S9D). We overnight fasted LC-LHA^hM4Di^ mice and controls, delivered CNO (0.3 μM) or vehicle directly into the LHA, and then measured food intake after 30 min. We observed no change in feeding between LC-LHA^hM4Di^ mice and controls administered CNO or vehicle into the LHA (fig. S9D). Further, we observed no change in feeding in sated mice using another strategy in which cre-dependent halorhodopsin (eNpHR3.0-EYFP) or EYFP was expressed in the LC of *Dbh^cre^* mice (fig. S9E) (*32*). Collectively, our opto- and chemogenetic studies demonstrate that activating the LC-LHA pathway suppresses feeding but inhibiting the pathway does not promote feeding.

## DISCUSSION

It is well established that LC-NE neurons promote alerting and orienting to salient external stimuli (*1*, *2*, *45*–*47*), but their involvement in the regulation of feeding remains less understood. In the current study, we found that, in contrast to their response to sensory stimuli and stressors (*4*, *5*, *48*, *49*), endogenous activity of LC-NE neurons is suppressed during feeding. Satiety attenuated the magnitude of LC-NE responses during feeding and to flashes of light. Further, we found that chemogenetic activation of LC-NE neurons suppressed ingestive behavior without altering metabolism. Further, we found that activation of LC-NE neurons reduced circadian food intake with a modest weight loss (2% loss). Using optogenetics, we found that feeding is suppressed when LC-NE neurons are activated by either a brief stimulation paired with feeding or stimulation over a longer duration. Chemogenetic inhibition of LC-NE neurons did not promote feeding. This finding is consistent with prior results showing that LC-NE activation promotes stress-like anxiety responses, but inhibition has no effect on baseline anxiety (*33*). Last, we found that the LC-LHA circuit is sufficient, but not necessary, for the suppression of feeding.

Our in vivo photometry experiments demonstrate that the activity pattern of LC-NE neurons is dynamically modulated during the consummatory sequence, with initial activation during food approach followed by a suppression of LC activity during consumption. This native pattern of LC-NE activity to food is unlikely attributed to changes in sleep, learning, stress, or anxiety, as our experimental mice were habituated to all aspects of the assay and were tested during the portion of the circadian cycle when they are the most active. Instead, the approach-related LC response we observed likely reflects an appetitive response to food in hungry mice. This finding is in agreement with prior electrophysiological studies that show that burst firing of LC neurons is associated with Pavlovian appetitive behavior in monkeys (e.g., innate, nonreinforced lipping) and operant responding (e.g., bar release for liquid reward) (*12*, *50*). In our open-loop optogenetic experiments, we observed that longer-term stimulation of LC-NE neurons tended to increase behaviors related to food seeking (e.g., increased entries and time spent in the food zone), although these effects were not statistically significant, indicating that longer-term stimulation of LC-NE has a limited effect on approach behaviors. Our closed-loop optogenetic studies demonstrate that discrete excitation of LC-NE neurons during consumption attenuated food intake, suggesting that transient inhibition of LC-NE activity is a regulatory mechanism naturally involved in feeding. Given that LC neurons are well known to modulate arousal (*1*, *45*, *51*, *52*), the decrease in LC activity we observed during feeding may reflect a decrease in arousal and/or disengagement with external sensory inputs to facilitate consummatory behavior.

Our findings demonstrate a previously unrecognized role for satiety in modulating endogenous LC responses during feeding. As mice ate more, the increase in LC activity during food approach was attenuated, a modulatory effect on LC-NE neurons that may be involved in the reduced salience of food as mice become sated. The contrasting decrease in LC activity during consumption was attenuated as mice ate to satiety. The satiety-related suppression of LC-NE activity during feeding could redirect attention to the external environment as satiation develops, although additional research is needed to test this possibility. Further, in fasted mice, we observed an increase in visual-evoked LC-NE activity compared to ad libitum–fed mice. Our results demonstrate that fasting potentiates LC-NE responses to sensory stimuli and suggest that LC is involved in enhanced salience detection upon fasting, which may potentially aid in food seeking and self-preservation during foraging. This potential interpretation is congruent with integrative theories of LC-NE neurons, which describe that this modulatory system is designed to optimize behavioral performance to a changing environment [reviewed in (*1*, *2*, *3*, *45*, *53*)]. Future studies will be required to directly test whether LC-NE responses to sensory stimuli change as a function of acute satiety in the regulation of foraging behavior and are needed to define the underlying mechanisms and circuits involved.

Recently, it has been shown using in vivo calcium imaging that a subpopulation of glutamatergic neurons in the peri-LC (peri-LC^VGLUT2^) is inhibited during consumption in a manner attenuated by satiety state (*54*), mimicking the activity patterns we observed in LC-NE neurons. It will be important for future studies to map the functional connectivity between LC-NE neurons and neighboring and distal targets that orchestrate behavioral states involved in feeding regulation. A recent study found that fear-induced suppression of feeding is mediated by LC-NE neurons through a projection to the lateral PBN (*13*). A series of seminal studies established that exogenous delivery of NE suppresses feeding [for review, see (*55*)], and that this effect is mimicked by direct delivery of NE agonists into the LHA [(*56*–*58*), see also (*59*)]. However, the source of NE that modulates these functions remains unclear, as the LHA receives input from LC and A2 noradrenergic nuclei (*20*, *60*–*62*). In the current study, we found that the LC→LHA pathway suppressed feeding. This effect is likely due to the enhancement of aversion and anxiety-like behavior following stimulation of this pathway. It is well established that LHA cell types drive a variety of complex behaviors, including feeding, drinking, anxiety, and arousal (*63*–*68*). It remains unclear whether these behaviors are modulated by separate or distinct LC-LHA circuits.

Our data suggest that there are likely other LC circuits involved in the modulation of feeding. Notably, the magnitude of feeding suppression was smaller when we activated LHA-projecting LC terminals compared to when we activated LC-NE cell bodies. We also found that stimulating the LC-LHA pathway suppressed feeding when optical pulses were continuously delivered during the feeding assay (30 min), but not when transiently paired with consumption events (10 s). These divergent effects may be related to the cellular, synaptic, and molecular mechanisms that remain to be determined, including the downstream synapses engaged and the type and amount of norepinephrine and/or neuropeptide(s) released from LC terminals following optical stimulation.

In conclusion, we demonstrate that during feeding LC-NE activity is transiently suppressed, and that food intake can be attenuated by briefly activating LC-NE neurons. We also identified a previously unrecognized role for satiety in modulating endogenous LC responses during feeding and to a visual flash of light. Further, our data provide emerging insight into the neural circuitry of motivated behavior, revealing a dual role of the LC-LHA pathway in feeding suppression and negative affective behavior. In context with the broader LC literature (*1*, *45*, *51*, *52*, *69*), our findings suggest that LC-NE neurons are involved in the modulation of feeding by integrating both external cues (e.g., anxiogenic environmental cues) and internal drives (e.g., satiety).

## MATERIALS AND METHODS

### Animals

All procedures related to the use of animals were approved by the National Institute of Environmental Health Sciences (NIEHS) Animal Care and Use Committee and were in accordance with the National Institutes of Health *Guide for the Care and Use of Laboratory Animals*. Adult (>P60) male and female mice were used for all studies. *En1^cre^*, *Dbh^cre^*, *Dbh^Flpo^*, and *RC::FL-hM3Dq* mouse colonies (*20*, *24*, *32*) are maintained on a C57BL/6J background. Triple transgenic *En1^cre^; Dbh^Flpo^; RC::FL-hM3Dq* mice and littermate controls were generated by crossing *En1^cre^* to double transgenic *Dbh^Flpo^; RC::FL-hM3Dq* mice. Mice were maintained on a reverse 12-hour light/12-hour dark cycle with lights off at 8 a.m. All experiments occurred during the dark period of the circadian cycle. Mice had ad libitum access to water and standard food (NIH-31, Harlan, Madison WI), except when mice underwent overnight food deprivation before the FI test and photometry recordings where noted. Mice were group-housed unless surgical implants or if CNO dosing by drinking water required single housing.

### Tissue collection

Adult mice were deeply anesthetized with sodium pentobarbital and perfused transcardially with phosphate-buffered saline (PBS) followed by 4% paraformaldehyde in PBS (PFA/PBS). Brains were postfixed overnight by immersion in 4% PFA/PBS at 4°C. Following rinse in PBS, tissue was cryoprotected in 30% sucrose in PBS and embedded in tissue freezing medium (General Data Healthcare). Forty-micrometer free-floating coronal or sagittal brain sections were collected in PBS, transferred to a cryoprotectant, and stored at −80°C.

### Immunohistochemistry

For immunofluorescence staining, mCherry-expressing neurons were detected using rat anti-mCherry primary antibody (1:1000) and goat anti-rat Alexa Fluor 568 secondary antibody (1:1000). EGFP- and EYFP-expressing neurons and axons were detected using chicken anti-GFP primary antibody (1:10,000) and goat anti-chicken Alexa Fluor 488 secondary antibody (1:1000). tdTomato^+^ neurons were detected using rabbit anti-dsRed primary antibody (1:1000) and goat anti-rabbit Alexa Fluor 568 secondary antibody (1:1000) or rat anti-tdTomato primary antibody (1:2000) and goat anti-rat Alexa Fluor 568 secondary antibody. The noradrenergic identity of neurons was confirmed with rabbit anti–tyrosine hydroxylase (TH) primary antibody (1:1000) and either goat anti-rabbit 633 or goat anti-rabbit 488 secondary antibody (1:1000). Mouse anti-TH primary antibody (1:500) was also used with goat anti-mouse 488, goat anti-mouse 568, or goat anti-mouse 633 secondary antibody (1:1000). Fos was detected with rabbit anti-cFos primary antibody (1:250; Santa Cruz Biotechnology, sc-52) and goat anti-rabbit 633 secondary antibody (1:1000). NeuroTrace 435/455 blue fluorescent Nissl stain (1:50; N21479, Thermo Fisher Scientific) was used to visualize neurons. For immunoperoxidase staining, Fos was detected using rabbit anti-cFos primary antibody (1:2000; ab190289, Abcam) and biotinylated goat anti-rabbit secondary antibody (1:500; BA-1000, Vector Labs) in conjunction with a Vectastain Elite ABC kit and a DAB substrate kit (Vector Labs). Coverslips were applied using Vectashield hard-set mounting medium with or without 4′,6-diamidino-2-phenylindole (DAPI) (H-1400 or H-1500, Vector Labs) or Prolong Diamond Anti-Fade mounting medium (P36970, Invitrogen). Methods were performed as previously described (*20*, *24*). Tissues from the different treatment groups were processed concurrently using the same batch of antibodies for all immunofluorescent experiments. The antibodies used are summarized in table S1.

### Digital image processing

Images of immunofluorescently labeled sections were collected using Zeiss LSM780 or 880 inverted confocal microscopes (Carl Zeiss Inc., Oberkochen, Germany). When necessary, Zen Black 2012 Software (Carl Zeiss) was used to convert z-stacks to maximum intensity projections. Images were modified only by adjusting brightness and contrast across the entire image to optimize the fluorescence signal. Anatomical location was confirmed by reference to a mouse brain atlas (*70*).

To measure Fos in LC-NE neurons, images of fluorescently stained sections were acquired at 20× using Zeiss LSM780, and digital images were subsequently exported to MetaMorph (Molecular Devices, San Jose, CA). To measure Fos in the hypothalamus, bright-field images of DAB-stained brain sections were acquired at 40× using an Aperio AT2 slide scanner (Leica Biosystems Inc., Buffalo Grove, IL). Digital images were subsequently exported from Aperio Imagescope (Leica Biosystems) as an uncompressed *.tif file and opened in FIJI software v2 (*71*) for further analysis.

### Cell counts

To measure Fos in LC-NE neurons, quantification was performed on every fourth 40-μm coronal section in the locus coeruleus and included at least two to four sections. An experimenter blind to treatment group performed the quantification. MetaMorph software was used to manually select TH^+^ and EGFP^+^ cells individually, and then we used the automated count nuclei feature to identify Fos cells using the following settings: Approximate minimum width was 6 μm, and maximum width was 9 μm with intensity above background of 10 gray levels. To determine colocalization, the AND operation was used within the arithmetic process to count colocalized pixels that were ≥20 μm^2^; this size was large enough to ignore background noise but small enough to count overlap of individual cells. To confirm our ability to detect Fos expression, we used LC^hM3Dq^ mice treated with CNO (5 mg/kg, intraperitoneally) and vehicle; this control tissue was run in every immunohistochemistry assay.

To measure Fos in hypothalamic nuclei, quantification was performed on every fourth 40-μm coronal section in the lateral, dorsomedial, and ventromedial hypothalamus, and included at least two to four sections. An experimenter blind to treatment group performed the quantification. FIJI software was used to measure the region of interest and perform automated counting. The number of Fos^+^ neurons was normalized to mm^2^ area.

### Drugs

For behavioral experiments using transgenic expression of the DREADD, we used the higher CNO dose (5 mg/kg, intraperitoneally). Given that viral delivery results in much higher levels of DREADD expression, we used the lower dose of CNO (1 mg/kg, intraperitoneally) for behavioral experiments using viral strategies. CNO [National Institute of Mental Health (NIMH) Drug Supply Program] was administered 20 min before behavioral testing (*24*). For study of Fos expression, CNO (1 mg/kg, intraperitoneally) or vehicle was administered 2 hours before perfusion, and groups were treated identically (e.g., same experimenter for injection and same day of perfusion) and were not subjected to any other treatment (e.g., fasting) or behavioral assay before sacrifice. All compounds were injected at a volume of 0.1 ml/10 g body weight. Drugs were dissolved in dimethyl sulfoxide (DMSO; <3%) and brought to volume using 0.9% physiological saline. For oral CNO administration, the doses selected were based on previously published papers (30 and 100 μg/ml) (*72*–*74*). CNO water was prepared fresh daily by dissolving in 0.5% DMSO and brought to volume using reverse osmosis deionized (RODI) water. For in vivo microinfusion studies, bilateral intra-LHA microinjection of CNO (0.3 μM) was administered 30 min before testing at a dose previously shown to modulate feeding behavior upon local activation of DREADD-expressing neural circuits (*75*). Microinjections of CNO were delivered in bubbled (95% O_2_ and 5% CO_2_) artificial cerebrospinal fluid (aCSF; containing 182 mM sucrose, 20 mM NaCl, 0.5 mM KCl, 1 mM MgCl_2_-6H_2_0, 1.2 mM NaH_2_PO_4_-H_2_0, 26 mM NaHCO_3_, and 10 mM glucose) at a volume of 100 nl at a rate of 100 nl/min through an internal bilateral cannula.

### Viral preparation

We generated an Flp-dependent GCaMP6f AAV construct for photometry experiments. pAAV-Ef1a-fDIO-EYFP (Addgene #55641) was digested with Asc I and Nhe I to remove the EYFP complementary DNA (cDNA), and GCaMP6f cDNA was obtained from pGP-CMV-GCaMP6f (Addgene #40755) (*76*) digested with Not I and Bgl II restriction enzymes. After incompatible single-strand overhangs were filled in to generate blunt ends, the two restriction fragments were ligated to produce plasmid pAAV-Ef1a-fDIO-GCaMP6f. To generate pAAV-Ef1a-fDIO-tdTomato, a tdTomato cDNA was isolated from pRSET-tdTomato (provided by R. Tsien) and cloned into pAAV-Ef1a EYFP digested with Asc I and Nhe I. To generate pAAV-Ef1a-fDIO-ChrimsonR-tdTomato, a ChrimsonR-tdTomato fusion cDNA was isolated from pAAV-Syn-FLEX-rc[ChrimsonR-tdTomato] (Addgene #62723) and cloned into pAAV-Ef1a-fDIO EYFP digested as above. The viruses and titers that were used are summarized in table S2.

### Surgery

Mice were anesthetized using 4% isoflurane and placed in a stereotaxic frame (Kopf Instruments, model 900) equipped with a digital micromanipulator (Harvard Apparatus, Holliston, MA). Anesthesia was maintained with 0.5 to 2% isoflurane and/or a cocktail of ketamine/xylazine (100/7 mg/kg, intraperitoneally). Bupivicaine (270 μg in 0.1 ml) was injected locally beneath the scalp before incision. For viral injections, 500-nl volume was delivered at the rate of 100 nl/min using a Neuros syringe (models 7001 and 7002, parts #65458-01 and #65459-01, Hamilton, Reno, NV) or 26-gauge needle attached to a microsyringe and pump (UMP3 UltraMicroPump, WPI, Sarasota, FL). Needles were left in place for 5 min after infusion to minimize backflow of the virus upon withdrawal of the needle. To minimize postoperative pain, all mice received the analgesic buprenorphine SR (1 mg/kg, subcutaneously). Mice were given a minimum recovery time of 4 weeks (for LC soma experiments) or 6 weeks (for LC fiber experiments) to allow sufficient time for viral infection, genetic recombination, and gene expression before behavioral measurements. See the Supplementary Materials for additional experimental details.

### LC-NE photometry recordings

In vivo optical recordings of GCaMP6f, tdTomato, and EGFP fluorescence intensities were measured in LC-NE neurons using a custom-built fiber photometry system, as previously described (*18*, *19*). Excitation light (488 nm, 20-mW continuous wave laser; OBIS 488LS-20, Coherent Inc.) was launched into a fluorescence cube (DFM1, Thorlabs), reflected by a dichroic mirror (ZT488/561rpc-UF1, Chroma), and focused by an achromatic fiber port (PAFA-X-4-A, Thorlabs) onto the core of a multimode patch cable (M83L01 was used for 200-μm core fiber probes, and M61L01 was used for 105-μm core fiber probes; Thorlabs). The distal end of the patch cable was connected to an optical fiber probe made with either a 200-μm-core-diameter, 0.39–numerical aperture (NA) multimode fiber (FT200EMT, Thorlabs) or a 105-μm-core-diameter, 0.22-NA multimode fiber (FG105LCA, Thorlabs) and a ceramic ferrule with a 1.25-mm outer diameter (MM-CON2007-2300 or MM-CON2010-1270-2-WHT, Precision Fiber Products) by a ceramic sleeve (SM-CS125S, Precision Fiber Products Inc.). The power of the excitation light measured at the tip of the implantable fiber probe was adjusted to 70 μW. Emitted fluorescence light was collected by the same optical fiber probe and patch cable, passed through the same dichroic mirror, and filtered through an emission filter (ZET 488/561m) before it was collected by a fiber port (PAF2S-11A, Thorlabs) and launched into a spectrometer (QE Pro-FL, Ocean Optics Inc.) through a multimode patch cable (M200L02-A, 0.22 NA, AR-coated, 200/240-μm core/cladding; Thorlabs). Time-lapse fluorescence emission spectra were visualized using Ocean View version 1.5. The spectrometer and camera (Basler, acA1300-60gm) were triggered using a Doric TTL pulse generator (OTPG_4, Neuroscience Studio Software, Doric Lenses Inc., Quebec, Canada). Mice were given at least 1 week for surgical recovery and then were habituated for several days (20 min/day) to being tethered to optical patch cables while in the testing arena (30 cm by 30 cm by 35.5 cm, Phenotyper, Noldus Information Technology Inc., Leesburg, VA, USA).

### Photometry data analysis

To quantify GCaMP6f emission and to separate fluorescence overlap between GCaMP6f and tdTomato, all raw emission spectra data were passed through a spectral linear unmixing algorithm written in R, as described previously (*18*). To control for movement artifacts in the fluorescence signal (e.g., photon loss caused by tissue movement or bending of the fiber during mouse movement), the unmixed GCaMP6f coefficients were normalized to unmixed tdTomato coefficients to generate GCaMP6f/tdTomato fluorescence ratio values that were converted to *z* scores. In a subset of mice without tdTomato expression (fig. S5), intensities of GCaMP6f or GFP emission were converted to fluorescence *z* scores. See the Supplementary Materials for additional details on the analysis.

### Behavioral experiments

#### Chemogenetic activation of LC-NE neurons during feeding

##### FI test

To motivate feeding, mice were fasted a day before the FI test (*77*). Food-deprived mice were allowed 20 min to explore a two-compartment arena (25 cm by 25 cm by 25 cm) that contained two petri dishes (60 mm by 15 mm) located on opposite corners. One dish contained standard food (NIH-31), and the other dish was empty. The dish location was randomly assigned and counterbalanced for each treatment group. For drug experiments, mice received CNO (5 mg/kg, intraperitoneally) (*24*) or vehicle ~20 min before the FI test. An experimenter blind to treatment recorded food intake. Mice were returned to ad libitum food after testing.

##### Labmaster feeding and metabolism

Mice were single-housed and allowed 4 days to acclimate to the Labmaster cages (23 cm by 13 cm by 1 cm) (TSE Systems, Bad Homburg, Germany). Following habituation, mice received water that contained vehicle or CNO (30 or 100 μg/ml) on alternate days, as used previously (*72*–*74*). At the same circadian time each day (CT 10 and 11), food and water were replenished, and mice were weighed by an experimenter. Food spillage was also monitored and measured by an experimenter, wherein large spillage events (>1 g in 15 min) were subtracted from total intake on rare occasions. Ad libitum food and drink was provided during the entire experiment. Measures of food and drink intake and gas exchange were collected every 15 min. Meal number and size were measured using the sequence meal analysis setting, whereby all meals were recorded chronologically to evaluate single feeding episodes. Meals started when food consumption was greater than 0.02 g and ended when consumption ceased for at least 5 min (*78*, *79*). For metabolic measures, O_2_ and CO_2_ were sampled for a 3-min period once every 15 min. Flow and sample rates were held at 0.3 and 0.25 liters/min, respectively, as determined by appropriate variation between the sample and reference cage. Respiratory exchange rate (RER) was derived by indirect calorimetry using the quotient VCO_2_/VO_2_. Energy expenditure (EE) was derived by the abbreviated Weir equation, EE = (3.941*VO_2_) + (1.106*VCO_2_). Metabolic values were normalized to the daily body weights (metabolic values/body weight). Daily cumulative intake and averages of gas exchange were assessed for each 24-hour drug-dosing day. Circadian data were summarized over three periods: early lights OFF (ZT11 to ZT20), lights ON (ZT20 to ZT8), and late lights OFF (ZT8 to ZT10). Data were collected using Labmaster version 2.6.9.13409 (TSE Systems) and analyzed by an experimenter blind to treatment.

### Optogenetic stimulation of LC-NE cell bodies or LC-LHA terminals during feeding

LC^ChrimsonR^ mice and LC^tdT^ controls were briefly anesthetized (4% isoflurane) to connect optical implants targeting the bilateral LC or LHA to patch cables [0.37 NA cable measuring 0.5 or 0.6 m in length, SBP(2)-200/220/900-0.37_0.45_FCM-2xMF1.25]. Mice were given 20 min to recover in their homecage. During the experiment, optical stimulation (560 nm, 10 Hz, 10-ms pulse width, and 6- to 7-mW total power from patch cable tip) was delivered using the Prizmatix Dual Channel Optogenetics Lime Green light-emitting diode (LED). Behavior was video-recorded using a Basler (acA1300-60gm) or Logitech (C930e) camera and analyzed by an experimenter blind to treatment group.

#### Open-loop optogenetics during the FI test

All mice were habituated to being tethered in the testing arena (20 cm by 20 cm by 25 cm) for 20 min on a day before testing. Mice were tested in a 30-min session while receiving 560-nm optical stimulation (10 Hz, 10-ms pulse width) or no stimulation. Food intake was recorded by an experimenter blind to treatment.

#### Closed-loop optogenetics during the FED pellet intake test

Before recordings, mice were habituated for several days to eating from the FED (*21*) in both the homecage and testing arena (Phenotyper, Noldus). Mice were then tested in a 60-min session, while they received pulses (10 ms) of optical stimulation (560 nm, 10 Hz) that were triggered upon pellet retrieval and terminated 10 s later. Thus, this protocol selectively delivered optical stimulation during discrete feeding events that were spontaneously initiated by the mice. Pellet retrieval time stamps were obtained from EthoVision, and retrieval and consumption events were confirmed by visual inspection of the video files. For quantification of other feeding behaviors, an experimenter blind to treatment condition used the manual score feature in EthoVision v15 to mark each experimental video file for precise time frames in which the (i) mouse began consuming a pellet and (ii) mouse dropped a pellet. “Consumption start” was defined as the first video frame in which a mouse brought a pellet up to its mouth. Consumption events that were difficult to see because of the positioning of the mouse or where the pellet was dropped before consumption were not scored for this measure (<10% of total consumption events for all mice). “Dropped pellets” was defined as any pellet that was dropped during the experiment, regardless of whether that pellet was eaten after dropping.

### Optogenetic stimulation of LC-LHA circuit during feeding and anxiety-related behaviors

LC-LHA^ChR2^ mice and LC-LHA^EYFP^ controls were briefly anesthetized (4% isoflurane) to connect the fiber-optic cannula to bilateral optical cables [0.48-NA cable measuring 0.5, 0.6, or 1 m in length with LC ferrule, BFP(2)_200/300/900-0.48_FCM-2xMF1.25]. While in the homecage, mice were given 20 min to recover before optical stimulation. For stimulation of LC fibers, mice received 10-Hz (10-ms pulse width) photostimulation (6- to 9-mW total power) using the Doric System (LEDFRJ 465 nm powered by LEDD driver, Neuroscience Studio Software, Doric Lenses), as previously described (*33*, *80*). Behavior was video-recorded using a Basler (acA1300-60gm) or Logitech (C930e) camera and analyzed by an experimenter blind to treatment group.

#### FI test

Mice were habituated to being tethered in the testing arena (20 cm by 20 cm by 25 cm) for 20 min the day before testing. Mice were tested in a 30-min session while receiving photostimulation (10 Hz, 10-ms pulse width). Food intake was recorded by an experimenter blind to treatment.

#### Elevated plus maze

Mice were placed in a “+” shaped maze (Stoelting Co.), as previously described (*24*). The maze had two open (35 cm by 5 cm, 15-mm lip) and two closed arms (35 cm by 5 cm by 15 cm) and was elevated 50 cm. Mice could explore for 5 min while receiving photostimulation (10 Hz, 10-ms pulse width). The time spent in the open arms (minutes) and ambulation (meters) were recorded using EthoVision v12-14. Open arm time is reported as (open arm time)/(open arm time + closed arm time) × 100.

#### Real-time place test

Mice were allowed 20 min to explore an unbiased two-compartment arena (50 cm by 50 cm by 25 cm), as described previously (*33*, *77*). Photostimulation (10 Hz, 10-ms pulse width) occurred upon entry into one compartment and persisted until the mouse exited the compartment. The stimulation compartment was randomly assigned and counterbalanced for each treatment group. The percentage of time spent in the photostimulation-paired compartment and ambulation were recorded by EthoVision v12-14.

#### Open field test

Mice were given 10 min to explore a clear plexiglass arena (45 cm by 45 cm by 30 cm) while they received photostimulation (10 Hz, 10-ms pulse width) (*24*). The time spent in the center of the arena and ambulation were recorded using EthoVision v12-14.

### Chemogenetic inhibition of LC-NE cell bodies or LC-LHA circuit during anxiety and feeding

#### Stress-induced anxiety in the OFT

Mice received CNO (1 mg/kg, intraperitoneally) approximately 30 min before being placed into a restraint tube (TV-150 STD, Braintree Scientific) for 30 min, as described previously (*33*). Immediately following restraint stress, mice were given 20 min to explore a novel open field arena (45 cm by 45 cm by 30 cm). The time spent in the center of the arena and total ambulation were recorded using EthoVision v15.

#### FI test

To motivate feeding, mice were overnight fasted before measuring food intake in a familiar arena. In LC^hM4Di^ cell body experiments, mice received CNO (1 mg/kg, intraperitoneally) or vehicle approximately 20 min before the FI test. For LC-LHA^hM4Di^ circuit experiments, mice were briefly anesthetized (4% isoflurane) to connect the internal cannulae to the guide cannulae and received bilateral local infusions of CNO (0.3 μM, 100 nl per side) (*75*) or aCSF (100 nl per side) at 100 nl/min through bilateral internal cannulae that projected 1.5 mm below the guide cannula (C235IS-5/SPC, P1 Technologies). Internal cannulae were left in place for 2 min to minimize backflow of the drug upon withdrawal. Mice were returned to their homecages for 30 min. Food intake was then measured after 30 min by an experimenter blind to treatment group.

### Optogenetic inhibition of LC-LHA circuit during feeding

LC-LHA^eNpHR^ mice and LC-LHA^EYFP^ controls were briefly anesthetized (4% isoflurane) to connect the fiber-optic cannula to bilateral polymer optical fibers (0.63 NA, 500 μm, 0.5-m length; Prizmatix), connected to an output port of a fiber-optic rotary joint (Prizmatix). Mice were given 20 min to recover in the homecage before testing. During the FI test (see the previous section for test details), mice received constant Lime-Green LED illumination (5-mW total power, Dual Channel Optogenetics-Lime-Green-LED module, Prizmatix). Food intake was measured for 30 min in free-feeding mice by an experimenter blind to treatment group.

### Statistical analysis

Analyses of variance (ANOVAs) and *t* tests were used to determine differences between groups. Bonferroni post hoc tests were used as appropriate. Significance was set at *P* < 0.05 for all analyses. All predictions were two-tailed unless otherwise stated to assess a priori predictions. All data are expressed as means ± SEM. For consistency across experiments, we present food intake in grams (e.g., FI test) or number of pellets consumed (e.g., FED studies). Further, we report percent change for experiments using repeated measure(s). To confirm that visual flashes increased peak responses of LC-NE activity, we used a one-tailed paired sample *t* test to assess a priori predictions. For quantification of Fos expression following LC^hM3Dq^ activation, we used one-tailed tests. Tukey’s strategy (1.5 interquartile range above or below the 25th or 75th percentile) was used for detection and removal of extreme outliers to avoid a type II statistical error. This conservative strategy identified the following outliers that were excluded from analysis: an LC^mCherry^ and LC^hM4Di^ mouse (open field) and LC-LHA^ChR2^ mouse (in baseline food intake). Analyses were conducted using GraphPad Prism 7 or 8 (GraphPad Software Inc.) and IBM SPSS for Windows, v21 (SPSS Inc., Chicago, IL). Statistics and sample size are listed in the figure legends.

## Notes

View/request a protocol for this paper from *Bio-protocol*.
