# Supplementary material for: Natural locus coeruleus dynamics during feeding
Source: Sci Adv. 2022 Aug 19;8(33):eabn9134. doi: 10.1126/sciadv.abn9134 (PMC9390985; doi:10.1126/sciadv.abn9134)
Supplement: Supplementary file 1 — Supplementary Materials and Methods Figs. S1 to S12 Tables S1 and S2 [file sciadv.abn9134_sm.pdf]

Science Advances
AAAS

Supplementary Materials for

Natural locus coeruleus dynamics during feeding

Natale R. Sciolino *et al.*

Corresponding author: Patricia Jensen, patricia.jensen@nih.gov

Sci. Adv. **8**, eabn9134 (2022)
DOI: 10.1126/sciadv.abn9134

This PDF file includes:

Supplementary Materials and Methods
Figs. S1 to S12
Tables S1 and S2

SUPPLEMENTARY MATERIALS AND METHODS

Table S1. Immunohistochemistry

The antibodies used are summarized below.

Antibody	Species	Dilution	Source	Catalog #	LOT #
mCherry	Rat	1:1000	Kerafast	EST202	105499, 032018
EGFP	Chicken	1:10,000<br>1:1000	Abcam	AB13970	236651-15
dsRed	Rabbit	1:1000	Clontech	632496	1612022
tdTomato	Rat	1:2000	Kerafast	EST203	Not available
TH	Rabbit	1:1000	Millipore	AB152	2493925
TH	Mouse	1:500	GeneTex	GTX10372	821901979,<br>821903616,<br>822101775
c-Fos	Rabbit	1:250	Santa Cruz	Sc-52	K1115
c-Fos	Rabbit	1:2000	Abcam	ab190289	GR289729-1
Alexa Fluor 568 anti-rat	Goat	1:1000	Invitrogen	A-11077	1853640
Alexa Fluor 488 anti-chicken	Goat	1:1000	Invitrogen	A-11039	1812246
Alexa Fluor 488 anti-rabbit	Goat	1:1000	Invitrogen	A-11034	1812166
Alexa Fluor 568 anti-rabbit	Goat	1:1000	Invitrogen	A-11036	1924788
Alexa Fluor 633 anti-rabbit	Goat	1:1000	Invitrogen	A-21071	2199090
Alexa Fluor 488 anti-mouse	Goat	1:1000	Invitrogen	A-11029	2306579
Alexa Fluor 568 anti-mouse	Goat	1:1000	Invitrogen	A-11031	685228
Alexa Fluor 633 anti-mouse	Goat	1:1000	Invitrogen	A-21052	1906490
Biotinylated anti-rabbit	Goat	1:500	Vector Labs	BA-1000	ZB0318

Table S2. Viral Preparation

The viruses used are summarized below.

Plasmid	Virus source	Packaging	Serotype	Titer
pAAV-Ef1a-fDIO-GCaMP6f (addgene ID 128315)	Jensen lab (NIEHS)	NIEHS Viral Vector core	AAV5	1.05 x 10^{13} vg mL^{-1}
pAAV-Ef1a-fDIO-tdTomato (addgene ID 128434)	Jensen lab (NIEHS)	NIEHS Viral Vector core	AAV5	6.46 x 10^{12} vg mL^{-1}<br>1.05 x 10^{13} vg mL^{-1}
pAAV-EF1a-fDIO-ChrimsonR-tdTomato (addgene ID 171027)	Jensen lab (NIEHS)	NIEHS Viral Vector core	AAV5	1.65 x 10^{13} vg mL^{-1}
pAAV-hSyn-DIO-hM4D(Gi)-mCherry (addgene ID 44362)	Roth lab (UNC-Chapel Hill)	Addgene	AAV5	1.8 x 10^{13} vg mL^{-1}
pAAV-hSyn-DIO-mCherry (addgene ID 50459)	Roth lab (UNC-Chapel Hill)	Addgene	AAV5	8.4 x 10^{12} vg mL^{-1}
pAAV-Ef1a-DIO hChR2(H134R)-EYFP	Deisseroth lab (Stanford)	UNC Viral Vector core	AAV5	7 x 10^{12} vg mL^{-1}
pAAV-Ef1a-DIO EYFP	Deisseroth lab (Stanford)	UNC Viral Vector core	AAV5	4.4 x 10^{12} vg mL^{-1}
pAAV-Ef1a-DIO-eNpHR3.0-EYFP	Deisseroth lab (Stanford)	UNC Viral Vector core	AAV5	3.1x 10^{12} vg mL^{-1}

Surgery

Surgery for Photometry Experiments. The locus coeruleus of *Dbh*^{Flpo} mice were injected with a 1:5 viral cocktail of *Flp*-dependent GCaMP6f (AAV5-fEf1a-fDIO-GCaMP6f) and tdTomato (AAV5-Ef1a-fDIO-tdTomato) using the following stereotaxic coordinates: −5.45 posterior; +/- 1.0 lateral; -3.75 ventral (mm from bregma). For experiments combining chemogenetics with photometry, both triple transgenic *En1*^{cre}*; Dbh*^{Flpo}*; R/C::FL-hM3Dq* (LC^{hM3Dq}) and double transgenic *Dbh*^{Flpo}*; R/C::FL-hM3Dq* (LC^{EGFP}) mice were used. The locus coeruleus of LC^{hM3Dq} mice were injected with a Flp-dependent GCaMP6f AAV, and a subset of these mice were also co-injected with a Flp-dependent tdTomato AAV at least 3 weeks before optical probe implantation. All mice were unilaterally implanted with optical probes above the LC (-5.45 posterior, ±0.85 lateral, -3.71 to -4.31 ventral, mm from bregma). Optical fiber probes were constructed by threading an optical fiber (200 µm, 0.39 NA, FT200EMT, Thorlabs; or 105 µm, 0.22 NA, Thorlabs) through a ceramic ferrule with a 1.25-mm OD (MM-CON2007–2300 or MM-CON2010-1270-2-WHT, Precision Fiber Products) and secured with heat-cured epoxy (Epoxy 353ND, Precision Fiber Products) and then polished and cleaved to length. Ventral placement of the optical fiber probe was guided by live fluorescence signal. During implantation of each probe, excitation light (470 nm, 760 mW mounted LED, M470L4, Thorlabs) was

launched into a fluorescence cube (DFM1, Thorlabs) containing an excitation filter (ET470/40x, Chroma) and beam splitter (T495lpxt, Chroma). Excitation light was then reflected into an achromatic fiber port (PAF2-A4A, Thorlabs) and collected by a 5-meter-long relay multimode patch cable (M72L05, 200-µm core with 0.39 NA, Thorlabs) connected with a 1-meter-long multimode patch cable (M83L01: 200-µm core with 0.39 NA or M61L01: 105-µm core with 0.22 NA) linked to an implantable ferrule containing the optical fiber by a ceramic sleeve (SM-CS125S, Precision Fiber Products Inc.). Excitation light from the implantable fiber was adjusted to ~70-µW. Using the same optical fiber and patch cable, emitted light was passed through an emission filter (ET500lp, Chroma) before collection by a multi-mode patch cable. Emitted light was then collected into a spectrometer (Ocean FX, Ocean Insight) and visualized using Ocean View version 1.6.7. Optical probes were adhered with epoxy (PFP 353ND, Precision Fiber Products, Inc.) and anchored to the skull using 3 skull screws, Metabond (S371, S398, and S396, Parkell Inc.) and dental acrylic (1406R and 1230CLR, Lang Dental Manufacturing Co. Inc.).

Surgery for ChrimsonR Optogenetic Experiments. *Dbh*^{Flpo} mice received bilateral LC injections of the Flp-dependent virus encoding ChrimsonR-tdTomato (AAV5-EF1a-fDIO-ChrimsonR-tdTomato) or tdTomato control (AAV5-EF1a-fDIO-tdTomato) using the following coordinates: -5.45 posterior, ±1.28 lateral, -3.65 mm ventral (mm from bregma)(*33*). Three weeks later, mice were implanted with bilateral fiber-optic cannula (200-µm, 0.39 NA, Thorlabs) angled 10° over LC using the following coordinates: -5.45 posterior, ±1.52 lateral, -3.50 to -3.65 ventral (mm from bregma) and angled 10° over the LHA using the following coordinates: -1.70 posterior, ±1.71 lateral, -4.81 to -4.86 ventral (mm from bregma). Ventral placement of the optical probe (200-µm, 0.39 NA, Thorlabs) was guided by live fluorescence signal, as described above in the *Surgery for Photometry Experiments* section. The implants were secured using Metabond and dental acrylic. We performed histology on every mouse to verify expression of virus and fiber placement. Importantly, we observed selective expression of ChrimsonR-tdTomato in TH+ locus coeruleus neurons, but no expression in other TH+ noradrenergic nuclei (e.g., A1, A2, A5) in all ChrimsonR mice.

Surgery for ChR2 and eNpHR Optogenetic Experiments. *Dbh*^{cre} mice received bilateral LC injections of the cre-dependent virus encoding ChR2-EYFP (AAV5-Ef1a-DIO-ChR2-EYFP), eNpHR (AAV5-Ef1a-DIO-eNpHR3.0-EYFP), or EYFP control (AAV-Ef1a-DIO-EYFP) using the following coordinates: -5.45 posterior, ±1.28 lateral, -3.65 mm ventral (mm from bregma)(*33*). During the same surgery, mice were implanted with bilateral fiber-optic cannula (MFC_200/250-0.66_5mm_ZF1.25_FLT, Doric Lenses Inc., Quebec Canada) angled 10° over the LHA using the following coordinates: -1.70 posterior, ±1.71 lateral, -4.82 ventral (mm from bregma)(*77*). The implants were secured using Metabond and dental acrylic. We performed histology on every mouse to verify expression of virus and fiber placement. Mice with selective expression of ChR2 or eNpHR in TH+ locus coeruleus neurons, but no expression in other TH+ noradrenergic nuclei (e.g., A1, A2, A5) were included for analysis.

Surgery for Gi-DREADD Experiments. *Dbh*^{cre} mice received bilateral LC injections of the cre-dependent virus encoding hM4Di (AAV5-hSyn-DIO-hM4Di-mCherry) or mCherry control (AAV5-hSyn-DIO-mCherry) using the following coordinates: -5.45 posterior, ±1.28 lateral, -

3.65 mm ventral (mm from bregma)(*33*). In the same surgery, mice were implanted with bilateral cannulae (C235GS-5-2.0/SPC, P1 Technologies, Roanoke VA) over the LHA using the following coordinates: -1.70 posterior, ±1.00 lateral, -3.45 ventral (mm from bregma)(*77*). Bilateral cannulae were secured using Metabond and dental acrylic. We performed histology on every mouse to verify expression of virus and cannula placement. Mice with selective bilateral expression of hM4Di in TH+ locus coeruleus neurons, but no expression in other TH+ noradrenergic nuclei (e.g., A1, A2, A5) were included for analysis.

LC-NE Photometry Recordings

Photometry Recordings for Sensory Experiments. A 1-s flash of light (set to 100-150 lux) was presented every minute during the recording session which lasted ~30-min. The light source (Dual Channel Optogenetics-Lime-Green-LED module, Prizmatix, Givat-Shmuel, Israel) connected to a polymer optical fiber (500-µm, NA 0.63, Prizmatix) that was positioned indirectly above the testing arena (Phenotyper).

Photometry Recordings for Feeding Experiments. Prior to recordings, mice were habituated for several days to eating from the feeding experimentation device (FED)(*21*) in both the homecage and testing arena (Phenotyper). The FED dispensed grain-based chow (25-mg pellets, Test Diet 5TUM) with a 60-s delay after retrieval of each pellet. This delay was used to space out pellets, which facilitated analysis of consumption responses during LC-NE calcium recordings in overnight fasted mice. Recordings collected with the FED lasted 60-min.

Photometry Recordings for Pharmacology Studies. Recordings lasted 35-min including 15-min prior to injection and 20-min post-injection. In all experiments, data were collected at 25-Hz from the camera and spectrometer (and FED when appropriate) and were synchronized using EthoVision (Noldus).

Photometry Data Analysis

Analysis of LC-NE Recordings During Feeding and Presentation of Light Flashes. We used RStudio (64-bit version 1.2.1335, RStudio, Inc., Boston, MA, USA) to run custom computer code (see *Code Availability* section) to align fluorescence ratio signals to relevant experimental events (i.e., pellet retrieval for feeding experiments, light flash for sensory experiments). The fluorescence ratio signal was assessed independently for each event around a 90-sec window for feeding experiments and 30-sec window for visual experiments. Each fluorescence ratio value was transformed into a z-score by subtracting the mean of a baseline period and dividing the SD of the baseline period. For feeding experiments, the 90-sec window was divided into four behavioral windows: baseline (45 to 30 sec before pellet retrieval), approach (1 to 0 sec before pellet retrieval), consumption (1 to 6 sec after pellet retrieval) and recovery (25 to 35 sec after pellet retrieval). Analysis periods were selected based on visual observation of behavior. In the feeding experiment, we compared LC responses during approach and consumption during the first and last ten pellets of the recording session to assess if LC activity varies as mice approached satiety.

For visual experiments, the 30-second window was divided into four behavioral windows: baseline (15 to 5 sec before light onset), pre-light (5 to 0 sec before light onset), light (0 to 1 sec), and post-light (5 to 15 sec after light onset). An average of the z-scores within the behavioral windows were reported and then averaged across events for each subject. Peak fluorescence ratio during the 1-sec light flash was found for each event and then averaged across events for each subject. We went back to our original dataset and identified two photometry recordings in fasted mice that had some visual events where fluorescence signal had a single frame rise and drop at the onset and offset of visual flashes. This is not normal calcium dynamics and suggests contamination of external light entering the recording fiber due to an unsecure mating sleeve. To isolate true fluorescence activity in an unbiased manner, the magnitude of single frame drops in fluorescence was subtracted from every data frame for every event where light leak was detected in the two recordings. Perievent rasters and associated example traces were plotted using Neuroexplorer 5.109 (64-bit version). All other traces were plotted using GraphPad Prism 7 or 8 (GraphPad Software Inc., La Jolla CA).

Analysis of LC-NE Recordings During Chemogenetic Experiments. For quantification of CNO or vehicle effects on LC activity, fluorescence z-scores were calculated using the 5 minutes prior to the experimenter entering for injection to determine the baseline fluorescence mean and standard deviation. The mean fluorescence z-scores across 5-min bins were then calculated. The photometry data collected during scruffing, and injection were excluded from quantification. These time points were manually marked in EthoVision v14 by an experimenter blind to treatment. Area under the curve of the fluorescence z-score traces during minutes 5-15 post injection were calculated in GraphPad Prism 8.

SUPPLEMENTAL FIGURES

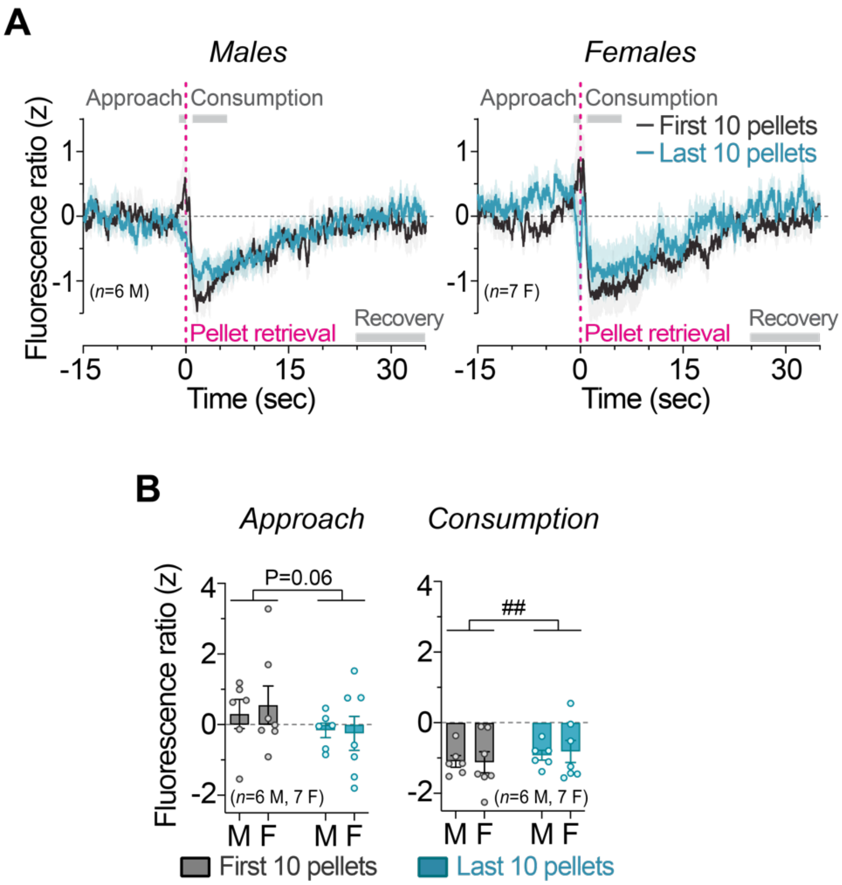

Fig. S1. Male and female mice have similar LC-NE activity to food. (A) Average z-score fluorescence ratio aligned to pellet retrieval in fasted LC$^{GCaMP/tdT}$ male (*Left*) and female mice (*Right*) during the first ten pellets and last ten pellets of the session. Data are mean ± SEM. *n*=6 males, 7 females. **(B)** Average z-score fluorescence ratio during feeding-related behaviors in male and female mice. Two-way ANOVA, satiety x sex interaction: approach ($F_{1,11}$=0.3106, P=0.5885) and consumption ($F_{1,11}$=1.111, P=0.3144). Main effect of sex: approach ($F_{1,11}$=0.02249, P=0.8835) and consumption ($F_{1,11}$=0.01336, P=0.9101). Main effect of satiety: approach ($F_{1,11}$=4.354, P=0.0610) and consumption ($F_{1,11}$=13.93, P=0.0033). $^{\#\#}P$< 0.01, first 10 vs. last 10 pellets. n.s., non-significant. Data are mean ± SEM. *n*=6 male and n=7 female LC$^{GCaMP6f/tdT}$ mice.

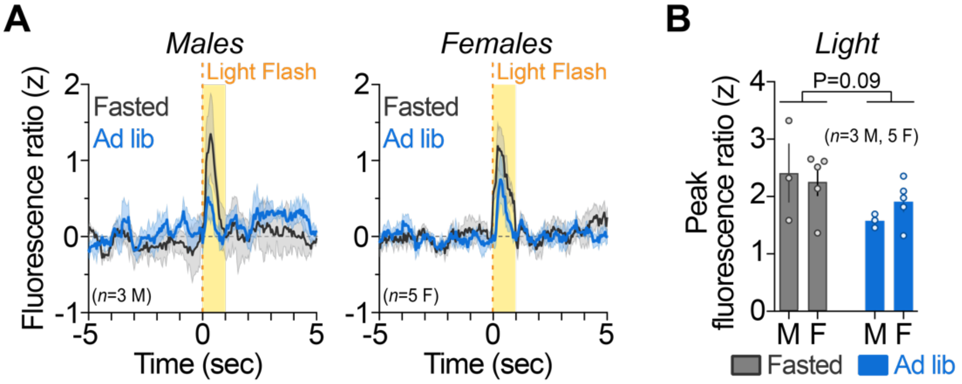

Fig. S2. Male and female mice have no significant differences in LC-NE activity to light flashes. (A) Average z-score fluorescence ratio aligned to visual flash in fasted $LC^{GCaMP/tdT}$ male (*Left*) and female mice (*Right*) during the fasted and ad libitum fed recordings. Data are mean ± SEM. *n*=3 males, 5 females. **(B)** Average peak fluorescence ratio during visual flashes in male and female mice. Two-way ANOVA, satiety x sex interaction: $F_{1,6}$=0.7245, *P*=0.4273. Main effect of sex: $F_{1,6}$=0.1287 *P*=0.7320. Main effect of satiety: $F_{1,6}$=4.225, *P*=0.0856. Data are mean ± SEM. *n*=3 male and n=5 female $LC^{GCaMP6f/tdT}$ mice.

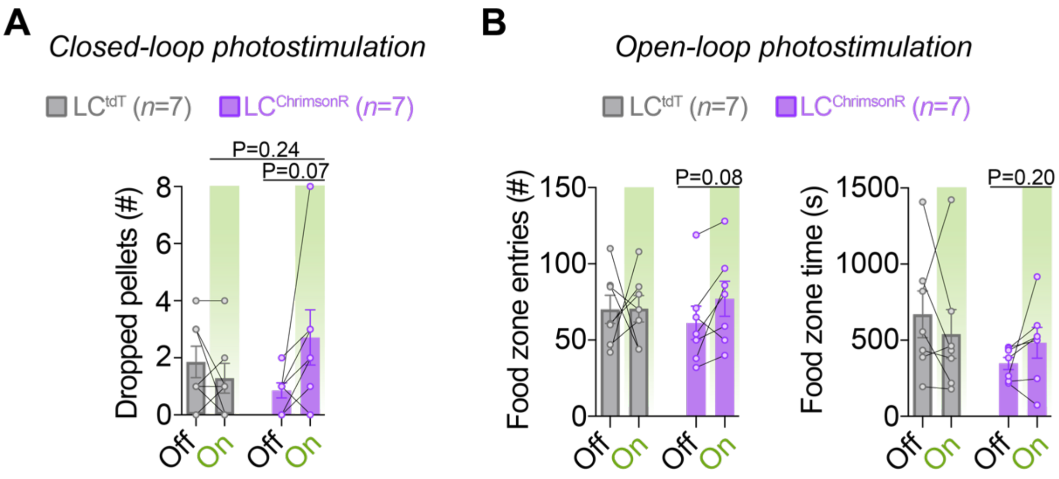

Fig. S3. Optogenetic activation of LC-NE neurons tended to increase the rate of pellet drops and food zone entries. (A) Feeding-related behaviors during the closed-loop optogenetic experiment wherein photostimulation (560-nm, 10-Hz, 10-ms pulses) was triggered upon pellet retrieval and occurred briefly for 10-sec during consumption from the feeding experimentation device (FED) in fasted mice. Two-way repeated measures ANOVA, stimulation x opsin interaction: dropped pellets ($F_{1,12}$=4.898, P=0.0470). Exact p-value shows the relevant Bonferroni post-hoc comparisons. n.s., non-significant. Data are mean ± SEM. n=7 LC^{tdT} mice, n=7 LC^{ChrimsonR} mice. **(B)** Food approach related behavior measured in the presence (On) or absence (Off) of open-loop photostimulation (560-nm, 10-Hz, 10-ms pulses) in fasted mice. Two-way repeated measures ANOVA, stimulation x opsin interaction: food zone entries (*Left*. $F_{1,12}$=0.6997, P=0.4192) and food zone time (*Right*. $F_{1,12}$=2.027, P=0.1800). Paired samples t-test: food zone entries (*Left*. t_6=2.121, P=0.0781) and food zone time (*Right*. t_6=1.451, P=0.1970). Data are mean ± SEM. n=7 LC^{tdT} mice, n=7 LC^{ChrimsonR} mice.

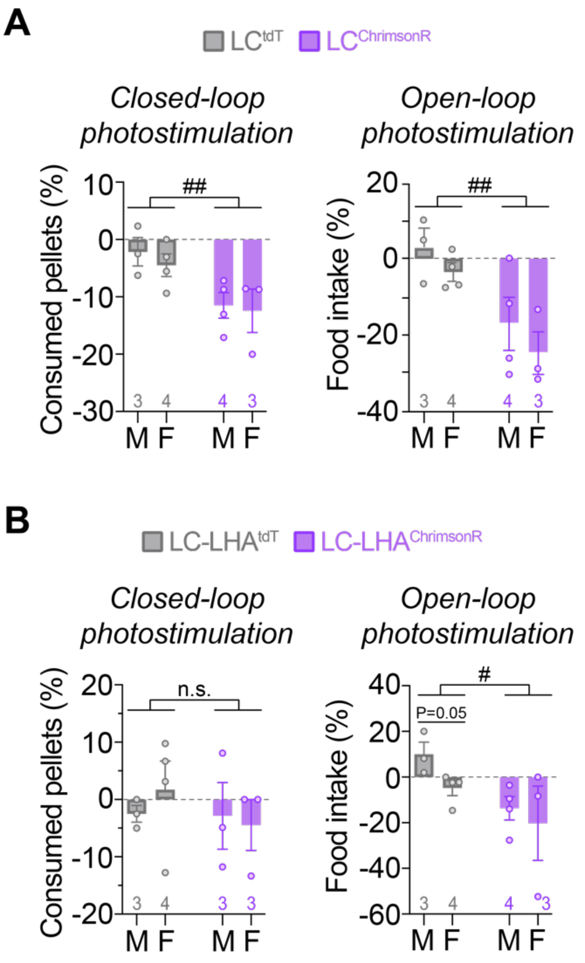

Fig. S4. Male and female mice have no significant differences in feeding suppression following optogenetic stimulation of LC-NE cell bodies and LC-LHA pathway. (A) Feeding during LC-NE cell body photostimulation as percent change from no stimulation during closed-loop (*Left*) and open-loop (*Right*) optogenetic experiments in fasted mice. Two-way ANOVA, photostimulation x sex interaction: closed-loop ($F_{1,10}$=0.07441, P=0.7906) and open-loop ($F_{1,10}$=0.01331, P=0.9104). Main effect of sex: closed-loop ($F_{1,10}$=0.4003, P=0.5411) and open-loop ($F_{1,10}$=1.687, P=0.2231). Main effect of photostimulation: closed-loop ($F_{1,10}$=11.09, P=0.0076) and open-loop ($F_{1,10}$=14.57, P=0.0034). $^{\#\#}P$< 0.01, ChrimsonR vs. tdT. n.s., non-significant. Data are mean ± SEM. n=3 male and n=4 female LC^{tdT} mice. n=4 male and n=3 female $LC^{ChrimsonR}$ mice. **(B)** Feeding during LC-LHA terminal photostimulation as percent change from no stimulation during closed-loop (*Left*) and open-loop (*Right*) optogenetic experiments in fasted mice. Two-way ANOVA, photostimulation x sex interaction: closed-loop ($F_{1,9}$=0.3804, P=0.5526) and open-loop ($F_{1,10}$=0.2692, P=0.6151). Main effect of sex: closed-loop ($F_{1,9}$=0.07634, P=0.7886) and open-loop ($F_{1,10}$=1.798, P=0.2096). Main effect of photostimulation: closed loop ($F_{1,9}$=0. 4764, P=0.5075) and open loop ($F_{1,10}$=5.971, P=0.0346). $^{\#}P$< 0.05, ChrimsonR vs. tdT. n.s., non-significant. Unpaired *t*-test: $LC\text{-}LHA^{tdT}$ males vs. females (*Right*, t_5=2.522, P=0.0531). Mean ± SEM. n=3 male and n=4 female $LC\text{-}LHA^{tdT}$ mice. n=3-4 male and n=3 female $LC\text{-}LHA^{ChrimsonR}$ mice.

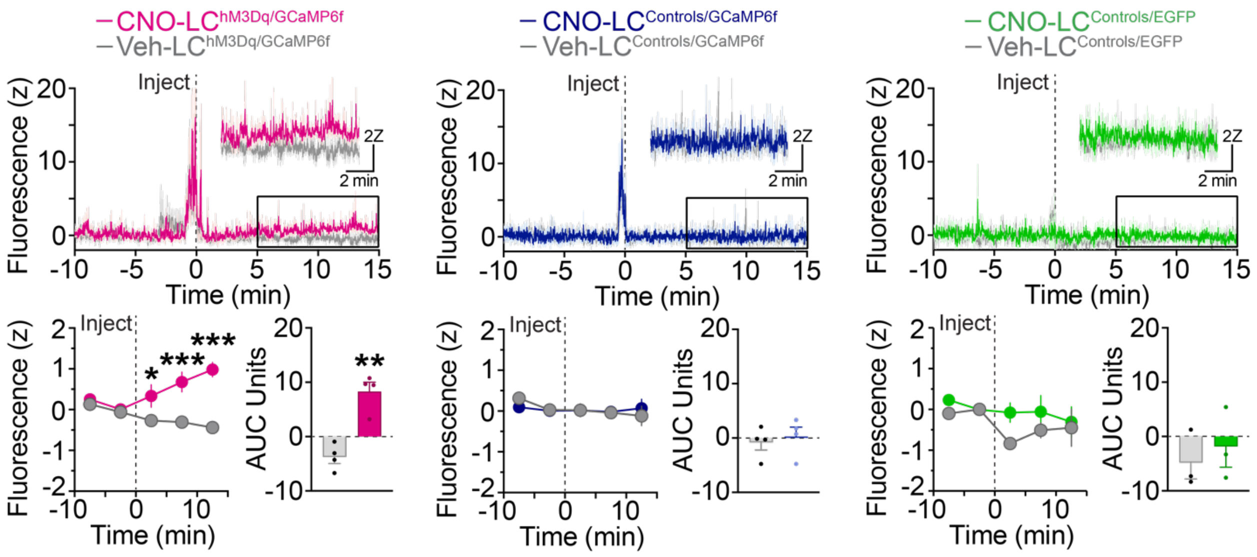

Fig. S5. Selective chemogenetic activation of LC-NE neurons. *Top.* Average z-score fluorescence ratio aligned to CNO (5 mg/kg i.p.) or vehicle injection in LC$^{hM3Dq/GCaMP6f}$ mice, littermate LC$^{Controls/GCaMP6f}$, and GCaMP6f-lacking littermate LC$^{Controls/EGFP}$. *Bottom left.* Average 5-min binned z-score fluorescence aligned to CNO or vehicle injection. Two-way repeated measures ANOVA, drug x time interaction: LC$^{hM3Dq/GCaMP6f}$ ($F_{4,12}$=11.99, P=0.0004), LC$^{Controls/GCaMP6f}$ ($F_{4,12}$=0.8213, P=0.5361), and LC$^{Controls/EGFP}$ ($F_{4,8}$=0.6928, P=0.6175). Bonferroni post-hoc test, ***P<0.01, *P<0.05 vs. vehicle. Data are mean ± SEM. n=4 LC$^{hM3Dq/GCaMP6f}$ mice, n=4 LC$^{Controls/GCaMP6f}$, and n=3 LC$^{Controls/EGFP}$. *Bottom right.* Area Under the Curve (AUC) from 5-min to 15-min following CNO or vehicle injection. Paired samples t-test: LC$^{hM3Dq/GCaMP6f}$ (t_3=10.22, **P<0.01), LC$^{Controls/GCaMP6f}$ (t_3=0.3990, P=0.7166), and LC$^{Controls/EGFP}$ (t_2=0.4699, P=0.6847). Data are mean ± SEM. n=4 LC$^{hM3Dq/GCaMP6f}$ mice, n=4 LC$^{Controls/GCaMP6f}$, and n=3 LC$^{Controls/EGFP}$.

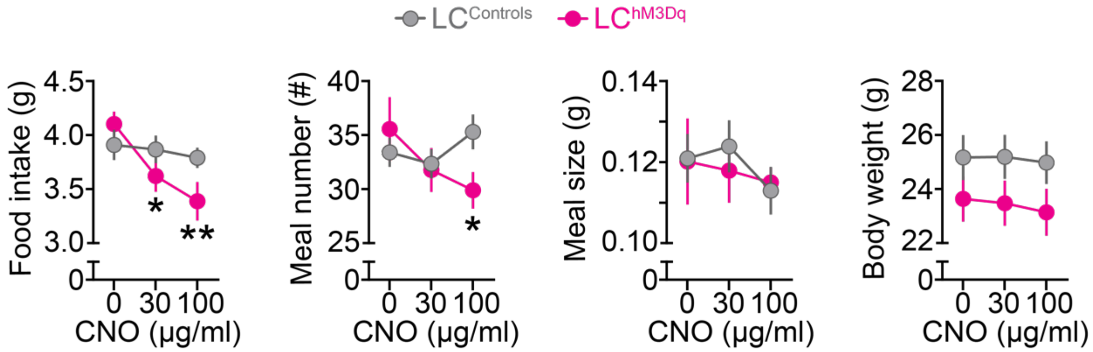

Fig. S6. Chemogenetic activation of LC-NE neurons suppresses feeding. Behavioral and metabolic measures in Labmaster automated homecage. Two-way repeated measures ANOVA, drug x genotype interaction: food intake ($F_{2,54}$=3.756, P=0.0297), meal number ($F_{2,54}$=4.518, P=0.0153), meal size ($F_{2,54}$=0.3750, P=0.6890), and body weight ($F_{2,70}$=2.559, P=0.0846). Bonferroni post-hoc test, **P<0.001, *P<0.01 vs. vehicle. Data are mean ± SEM. n=20 LC^{Controls}, n=9 LC^{hM3Dq} mice for all measures except body weight wherein n=17 LC^{hM3Dq} mice.

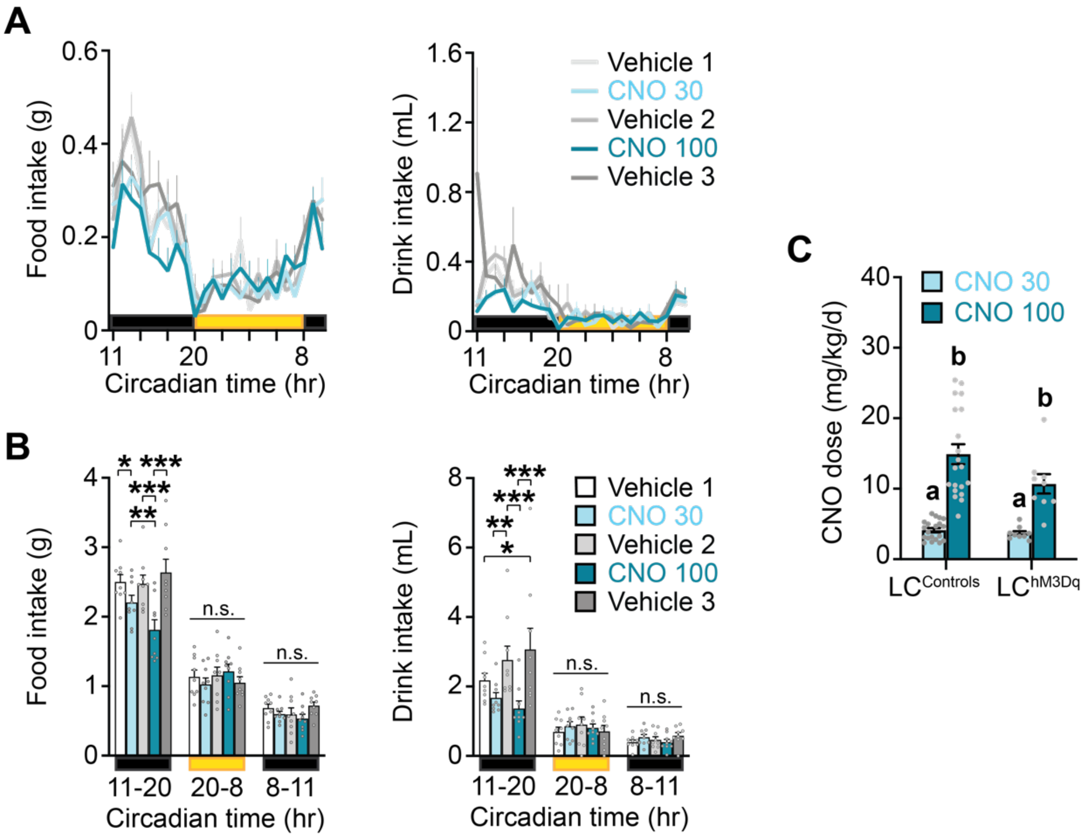

Fig. S7. Chemogenetic activation of LC-NE neurons suppresses feeding and drinking during the early, dark-phase of the light cycle in a reversible manner. (A) Circadian food and drink intake by LC^{hM3Dq} mice receiving CNO (30 and 100 μg/mL) in drinking water. **(B)** Average food and drink intake during the 24-hour circadian light cycle. Two-way repeated measures ANOVA, drug x time interaction: food intake ($F_{8,64}$=8.02, P<0.001) and drink intake ($F_{8,64}$=3.93, P<0.001). Bonferroni post-hoc test, ***P<0.001, **P<0.01, *P<0.05. n.s., non-significant. Data are mean ± SEM. n=9 LC^{hM3Dq} mice. CT, circadian time. Yellow shows circadian lights-on (CT 20-8). Black shows circadian lights-off, which was divided according to when drug was first introduced (CT 11-20; early dark) and later present (CT 8-11; late dark). **(C)** Average daily CNO dose by drinking water. Two-way repeated measures ANOVA, drug x genotype interaction: ($F_{1,27}$=3.995, P=0.0558). Main effect of drug: ($F_{1,27}$=87.96, P<0.0001), P<0.001 30 μg/mL (group a) vs. 100 μg/mL (group b). Data are mean ± SEM. n=20 LC^{Controls}, n=9 LC^{hM3Dq} mice.

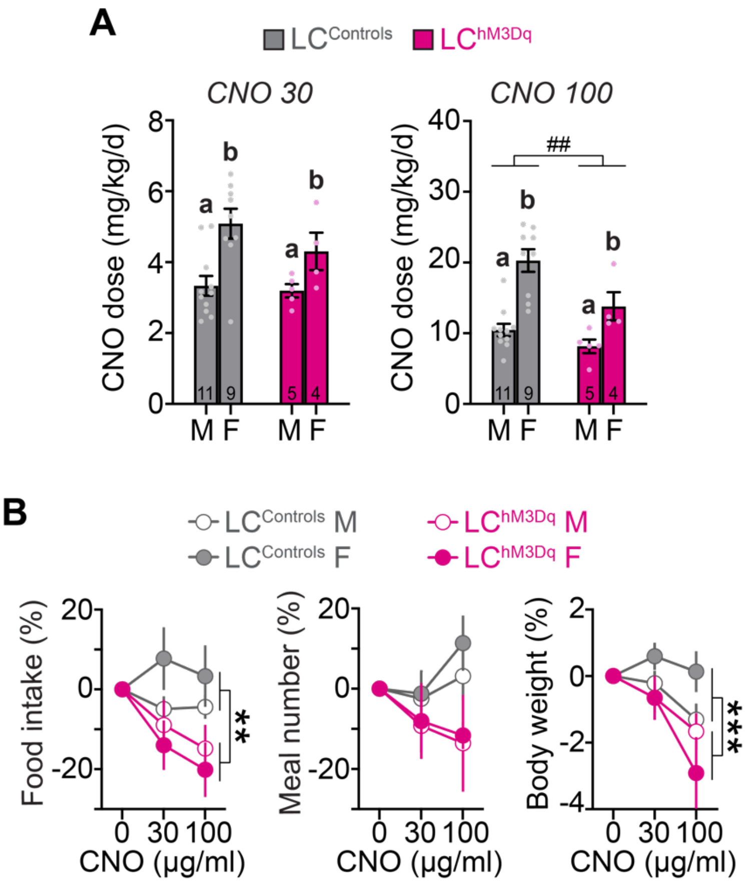

Fig. S8. Females drink more CNO water and receive an increased daily dose compared to males, but no significant differences in metabolic measures were observed between the sexes. (A) Average daily CNO dose by drinking water in male and female mice. Two-way ANOVA, sex x genotype interaction: CNO 30 µg/mL ($F_{1,25}$=0.6241, P=0.4370) and CNO 100 µg/mL ($F_{1,25}$=1.987, P=0.1710). Main effect of sex: CNO 30 µg/mL ($F_{1,25}$=12.46, P=0.0016) and CNO 100 µg/mL ($F_{1,25}$=27.39, P<0.001). Main effect of genotype: CNO 30 µg/mL ($F_{1,25}$=1.297, P=0.2656) and CNO 100 µg/mL ($F_{1,25}$=8.827, P=0.0065). P<0.01 males (group a) vs. females (group b). $^{\#\#}P$< 0.01, LC^{Controls} vs. LC^{hM3Dq}. Data are mean ± SEM. LC^{Controls} n=11 males, n=9 females. LC^{hM3Dq} n=5 males, n=4 females. **(B)** Behavioral and metabolic measures in the automated homecage in male and female mice. Three-way ANOVA, drug x genotype x sex interaction: food intake ($F_{2,50}$=1.061, P=0.3537), meal number ($F_{2,50}$=0.1057, P=0.8999), and body weight ($F_{2,66}$=3.055, P=0.0538). Drug x sex interaction: food intake ($F_{2,50}$=0.1892, P=0.8282), meal number ($F_{2,50}$=0.2332, P=0.7929), and body weight ($F_{2,66}$=0.2876, P=0.7510). Drug x genotype interaction: food intake ($F_{2,50}$=,3.937 P=0.0258), meal number ($F_{2,50}$=3.359, P=0.0428), and body weight ($F_{2,66}$=4.645, P=0.0130). Genotype x sex interaction: food intake ($F_{1,25}$=1.679, P=0.2069), meal number ($F_{1,25}$=0.05055, P=0.8239), and body weight ($F_{1,33}$=3.089, P=0.0881). Bonferroni post-hoc test, ***P<0.001, **P<0.01 vs. LC^{Controls} vs. LC^{hM3Dq} mice. Data are mean ± SEM. LC^{Controls} n=11 males, n=9 females. LC^{hM3Dq} n=5 males, n=4 females for all measures except body weight, wherein LC^{hM3Dq} males n=10 and females n=7.

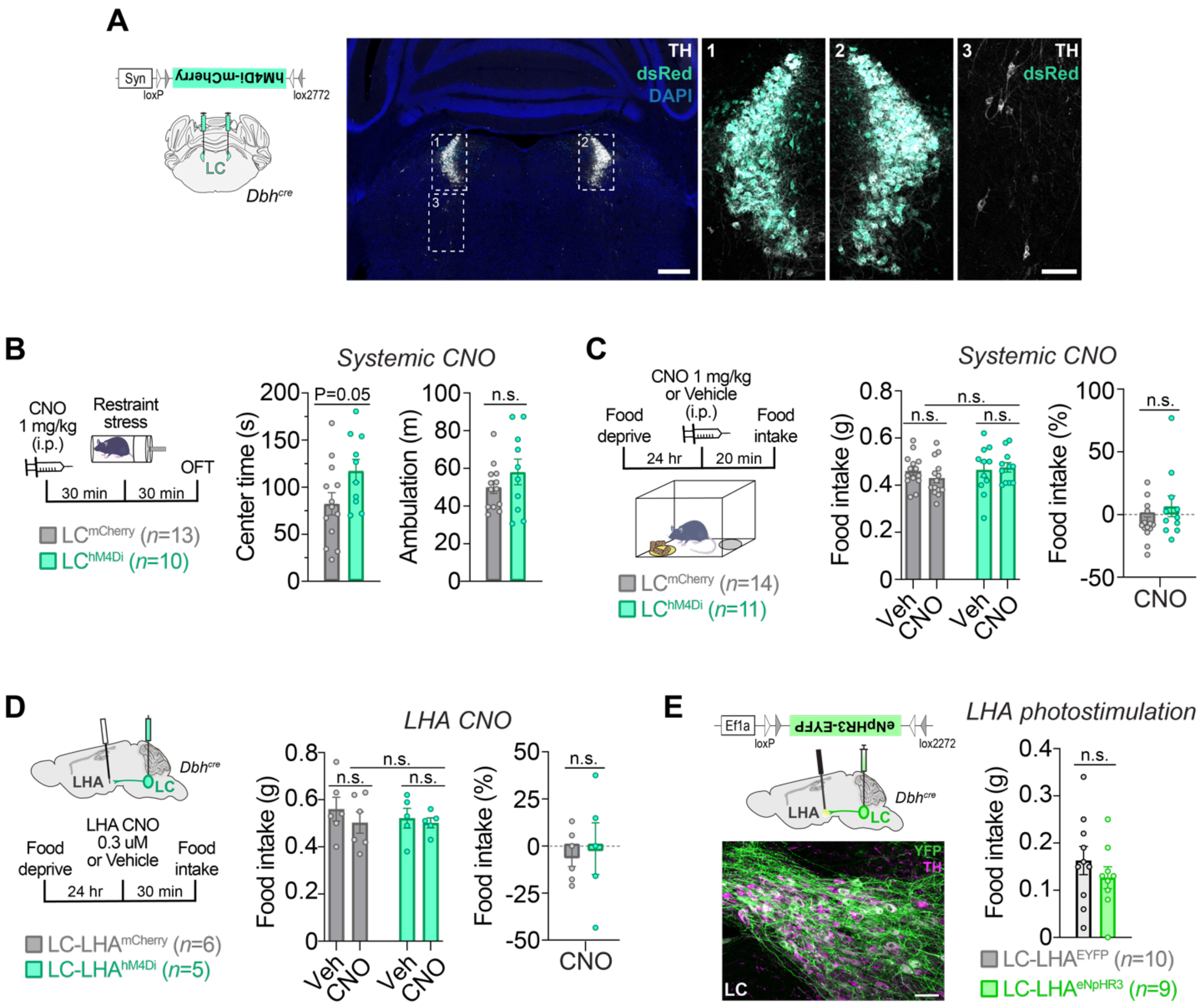

Fig. S9. Inhibition of either LC-NE cell bodies or LC-LHA pathway results in no significant change in food intake. (A) *Left.* Schematic illustration of a coronal mouse brain shows the location of cre-dependent AAV used to drive hM4Di-mCherry expression. *Right*. Immunofluorescent labeling shows expression of mCherry (dsRed, blue-green) in noradrenergic (TH, white) neurons of the bilateral LC (images 1 and 2) but not in noradrenergic ventral subcoeruleus (SubC, image 3) neurons in a coronal brain section from a LC^{hM4Di} mouse. Scale is 400-µm (brain slice) and 100-µm (high-magnification images). **(B)** *Left.* Timeline of stress-induced anxiety experiments. *Right***.** Average time spent in the center of the open field test (OFT) and ambulation following systemic CNO delivery and stress exposure. Unpaired samples *t*-test: center time (t_{21}=2.036, P=0.0545) and ambulation (t_{21}=1.139, P=0.2674). Data are mean ± SEM. n=13 LC^{mCherry} mice, n=10 LC^{hM4Di} mice. **(C)** Food intake (FI) following systemic delivery of CNO. *Left.* Timeline of FI experiments. *Middle***.** Average FI in fasted mice in CNO and vehicle-treated mice. Two-way repeated measures ANOVA, drug x effector interaction: $F_{1,23}$=1.540, P=0.2271. n.s., non-significant. *Right.* CNO-evoked food intake as a percent change from vehicle. Unpaired samples *t*-test, t_{23}= 1.442, P=0.1629. Data are

mean ± SEM. *n*=14 $LC^{mCherry}$ mice, *n*=11 LC^{hM4Di} mice. **(D)** *Left.* Schematic illustration of sagittal mouse brain shows the location of cre-dependent AAV used to drive hM4Di-mCherry expression and drug infusion cannulae. Timeline of intra-LHA delivery of CNO (0.3 μM) before the FI assay. *Middle***.** Average food intake following intra-LHA delivery of CNO or vehicle in fasted mice. Two-way repeated measures ANOVA, drug x effector interaction: $F_{1,9}$=0.1953, *P*=0.6689. n.s., non-significant. *Right.* CNO-evoked feeding as a percent change from vehicle. Unpaired samples *t*-test, t_9= 0.2822, *P*=0.7842. Data are mean ± SEM. *n*=6 $LC\text{-}LHA^{mCherry}$ mice, *n*=5 $LC\text{-}LHA^{hM4Di}$ mice. **(E)** *Left.* Schematic illustration of a parasagittal mouse brain shows the location of cre-dependent AAV used to drive eNpHR3.0-EYFP expression and fiberoptic probes. Immunofluorescent labeling shows eNpHR3.0-EYFP expression (EYFP, green) in noradrenergic (TH, magenta) locus coeruleus neurons in a parasagittal brain section from a $LC\text{-}LHA^{eNpHR3}$ mouse. Scale, 50-μm. *Right.* Average food intake during photostimulation for 30-min in *ad libitum* fed mice. Unpaired samples *t*-test: t_{17}=0.9491, *P*=0.3559. Data are mean ± SEM. *n*=10 $LC\text{-}LHA^{EYFP}$ mice, *n*=9 $LC\text{-}LHA^{eNpHR3}$ mice.

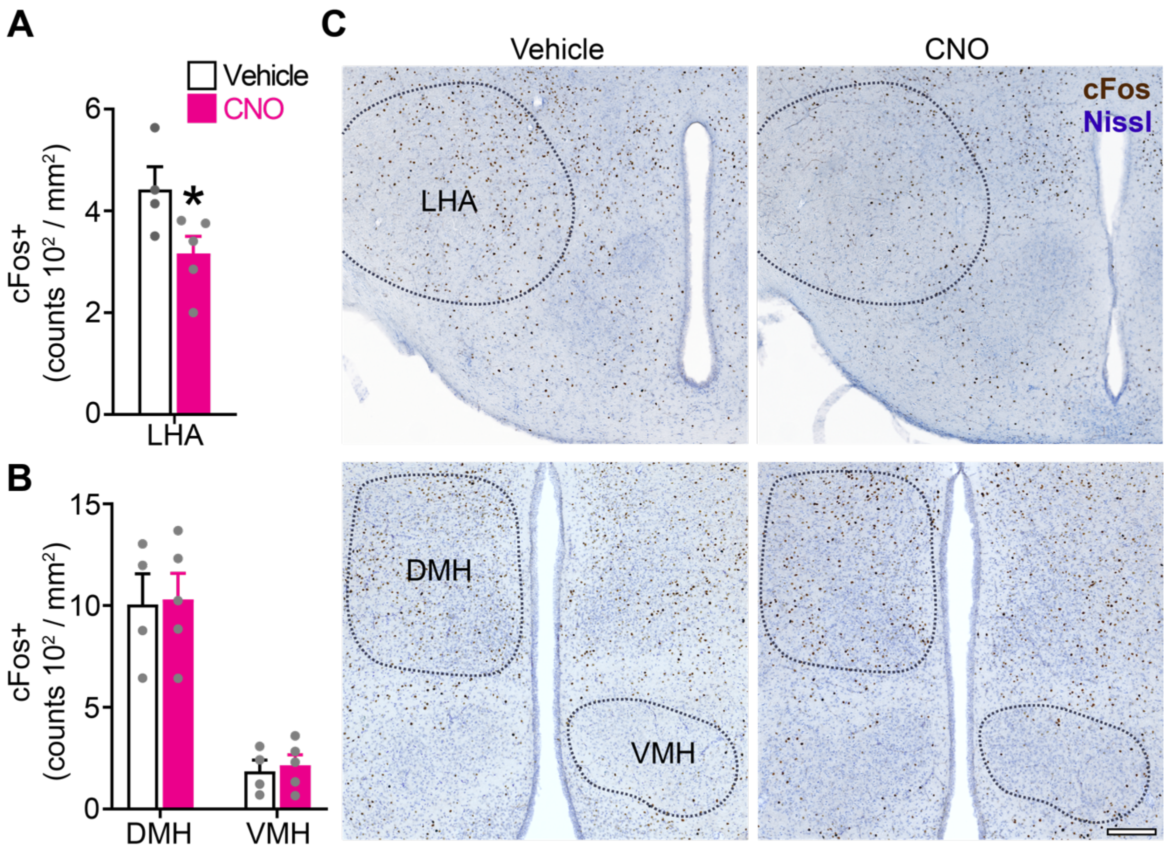

Fig. S10. Chemogenetic activation of LC-NE neurons reduces neuronal activity in the LHA. **(A-B)** Average Fos+ cell counts in the LHA of LC^{hM3Dq} mice. Unpaired samples *t*-test (one-tailed): LHA (**A**, t_7=2.302, *P<0.05), DMH (**B**, t_7=0.1281, P=0.4508), and VMH (**B**, t_7=0.3852, P=0.3558). Data are mean ± SEM. n=4 vehicle mice, n=5 CNO (1 mg/kg i.p.) mice. **(C)** Immunofluorescent labeling of coronal brain sections shows expression of Fos (brown) in hypothalamic nuclei (outlined) of LC^{hM3Dq} mice. Tissue was counterstained with Nissl (blue). Scale, 100-µm. LHA, lateral hypothalamus area. DMH, dorsal medial hypothalamus. VMH, ventral medial hypothalamus.

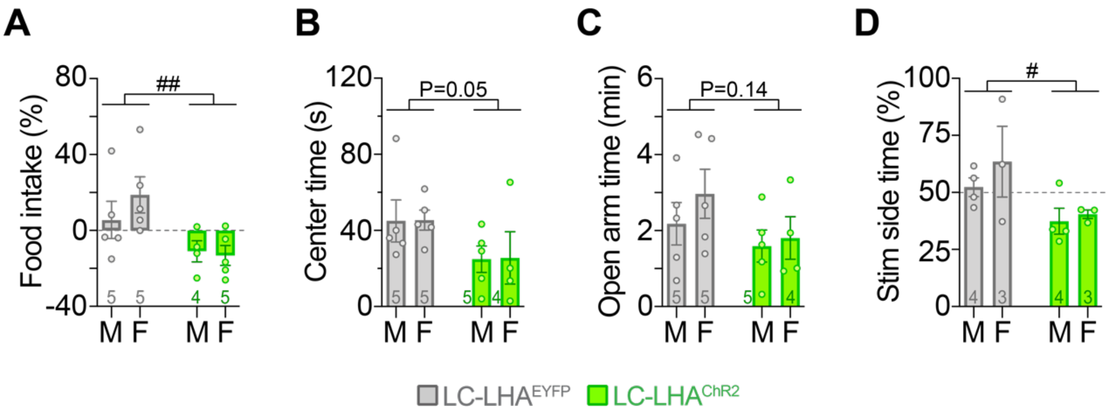

Fig. S11. Male and female mice have no significant differences in feeding suppression, anxiety-like behavior and aversion following optogenetic stimulation of the LC-LHA pathway. (A) Feeding during LC-LHA terminal photostimulation as percent change from no stimulation in fasted mice. Two-way ANOVA, photostimulation x sex interaction: $F_{1,15}$=0.9049, P=0.3566. Main effect of sex: $F_{1,15}$=0.4643, P=0.5060. Main effect of photostimulation: $F_{1,15}$=8.884, P=0.0093. **(B)** Center time in the OFT during photostimulation. Two-way ANOVA, photostimulation x sex interaction: $F_{1,15}$=0.0000, P=0.9924. Main effect of sex: $F_{1,15}$=0.002960, P=0.9573. Main effect of photostimulation: $F_{1,15}$=4.540, P=0.0501. **(C)** Open arm time in the EPM during photostimulation. Two-way ANOVA, photostimulation x sex interaction: $F_{1,15}$=0.2651, P=0.6142. Main effect of sex: $F_{1,15}$=0.8006, P=0.3850. Main effect of photostimulation: $F_{1,15}$=2.460, P=0.1376. **(D)** Percent time spent in the photostimulation-paired side of the RTPT. Two-way ANOVA, photostimulation x sex interaction: $F_{1,10}$=0.2736, P=0.6123. Main effect of sex: $F_{1,10}$=0.8464, P=0.3792. Main effect of photostimulation: $F_{1,10}$=6.027 P=0.0340. Data are mean ± SEM. LC-LHA^{EYFP} n=4-5 male and n=3-5 female mice. LC-LHA^{ChR2} n=4-5 male and n=3-5 female mice. $^{\#\#}P< 0.01$, $^{\#}P< 0.05$, LC-LHA^{ChR2} vs. LC-LHA^{EYFP}.

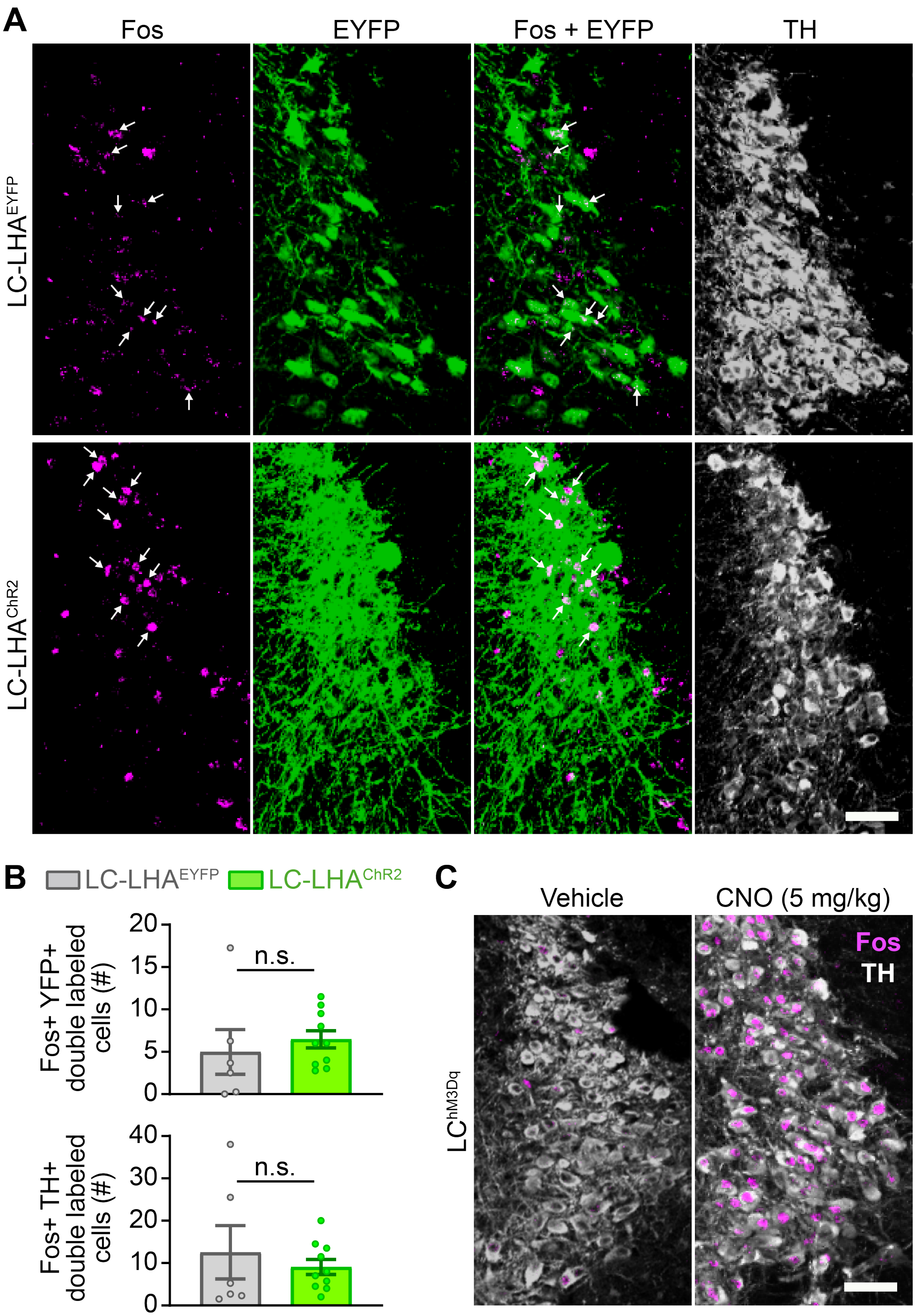

Fig. S12. Stimulation of LC-LHA projection terminals does not influence LC-NE cell body activity. (A) Immunofluorescent labeling of coronal brain sections shows co-expression of Fos (magenta) and EYFP (green) in noradrenergic (TH, white) locus coeruleus neurons of LC-LHA^{ChR2} mice and LC-LHA^{EYFP} controls. Arrows indicate location of double-labeled Fos+ YFP+ neurons in the locus coeruleus. Scale, 50-µm. **(B)** *Top.* Double-labeled Fos+

YFP+ neurons in the locus coeruleus. Unpaired samples *t*-test: t_{14}=0.6213, *P*=0.5444. *Bottom.* Double-labeled Fos+ TH+ neurons in the locus coeruleus. Unpaired samples *t*-test: t_{14}=0.6528, *P*=0.5244. Data are mean ± SEM. *n*=6 LC-LHA^{EYFP} mice, *n*=10 LC-LHA^{ChR2} mice. **(C)** Immunofluorescent labeling of coronal brain sections shows co-expression of Fos (magenta) in noradrenergic (TH, white) locus coeruleus neurons of vehicle-treated (negative control) and CNO-treated (positive control) LC^{hM3Dq} mice, confirming our ability to detect Fos expression. Scale, 50-µm.
